# PET-MRI biomarkers reveal efficacy of a novel NLRP3 inhibitor in Parkinson’s disease models

**DOI:** 10.1093/brain/awaf372

**Published:** 2025-10-16

**Authors:** Eduardo A Albornoz, Karine Mardon, Rajiv Bhalla, Vinod Kumar, Damion H R Stimson, Gary Cowin, Cedric S Cui, Mark S Butler, Ruby Pelingon, Richard Gordon, Rebecca C Coll, Kate Schroder, Reena Halai, Angus M MacLeod, Kim Matthews, Avril A B Robertson, Matthew A Cooper, Trent M Woodruff

**Affiliations:** School of Biomedical Sciences, Faculty of Health, Medicine and Behavioural Sciences, The University of Queensland, Brisbane 4072, Australia; Centre for Advanced Imaging, The University of Queensland, Brisbane 4072, Australia; Centre for Advanced Imaging, The University of Queensland, Brisbane 4072, Australia; School of Biomedical Sciences, Faculty of Health, Medicine and Behavioural Sciences, The University of Queensland, Brisbane 4072, Australia; Centre for Advanced Imaging, The University of Queensland, Brisbane 4072, Australia; Centre for Advanced Imaging, The University of Queensland, Brisbane 4072, Australia; School of Biomedical Sciences, Faculty of Health, Medicine and Behavioural Sciences, The University of Queensland, Brisbane 4072, Australia; The Institute for Molecular Bioscience, The University of Queensland, Brisbane 4072, Australia; The Institute for Molecular Bioscience, The University of Queensland, Brisbane 4072, Australia; School of Biomedical Sciences, Faculty of Health, Medicine and Behavioural Sciences, The University of Queensland, Brisbane 4072, Australia; Translational Research Institute (TRI), School of Biomedical Sciences, Faculty of Health, Queensland University of Technology, Brisbane 4102, Australia; The Institute for Molecular Bioscience, The University of Queensland, Brisbane 4072, Australia; The Wellcome-Wolfson Institute for Experimental Medicine, Queen’s University Belfast, Belfast BT9 7BL, UK; The Institute for Molecular Bioscience, The University of Queensland, Brisbane 4072, Australia; Inflazome Ltd, Welwyn Garden City AL7 1TW, UK; Sitala Bio Ltd., Shelford, Cambridgeshire CB22 1LD, UK; Inflazome Ltd, Welwyn Garden City AL7 1TW, UK; Inflazome Ltd, Welwyn Garden City AL7 1TW, UK; Sitala Bio Ltd., Shelford, Cambridgeshire CB22 1LD, UK; School of Chemistry and Molecular Biosciences, The University of Queensland, Brisbane 4072, Australia; Inflazome Ltd, Welwyn Garden City AL7 1TW, UK; Sitala Bio Ltd., Shelford, Cambridgeshire CB22 1LD, UK; School of Biomedical Sciences, Faculty of Health, Medicine and Behavioural Sciences, The University of Queensland, Brisbane 4072, Australia; Queensland Brain Institute, The University of Queensland, Brisbane 4072, Australia

**Keywords:** Parkinson’s disease, drug discovery, neuroinflammation, inflammasome, PET imaging

## Abstract

Parkinson’s disease is one of the fastest-growing neurodegenerative disorders, with no effective treatments to modify its progression. Microglial-driven neuroinflammation, mediated by NOD-leucine rich repeat and pyrin containing protein 3 (NLRP3) inflammasome activation, plays a key role in disease onset and progression. The NLRP3 inflammasome is upregulated in microglia from Parkinson’s disease patients and activated by oxidative stress and α-synuclein aggregates, triggering the release of pro-inflammatory mediators that contribute to neuroinflammation and neuronal death. MCC950, the first described specific NLRP3 inhibitor, has shown promise in Parkinson’s disease models but is limited by suboptimal pharmacokinetics and safety, hindering its clinical development.

Here, we developed a novel NLRP3 inflammasome inhibitor, MCC7840 (also known as Inzomelid or Emlenoflast), and utilized clinically relevant PET-MRI imaging biomarkers to assess its therapeutic efficacy in preclinical models of Parkinson’s disease.

MCC7840 inhibited NLRP3 in human and mouse microglia with nanomolar potency, while demonstrating improved systemic exposure, half-life, brain permeability and bioavailability compared with MCC950. In a murine NLRP3 gain-of-function model of Muckle–Wells syndrome, MCC7840 effectively inhibited mortality and demonstrated superior potency compared with MCC950. Chronic oral administration of MCC7840 protected against neuroinflammation, motor deficits and dopamine loss in both 6-hydroxydopamine and preformed α-synuclein fibril mouse models of Parkinson’s disease. Radiotracer imaging of multiple PET markers in the same mouse revealed that MCC7840 attenuated neuroinflammation (translocator protein ligand; ^18^F-DPA-714), preserved dopamine uptake (fluorodopa; ^18^F-FDOPA), mitigated dopamine transporter (DAT) loss (DAT ligand; ^18^F-FBCTT) and reduced blood–brain barrier leakage (gadolinium contrast MRI). Notably, MCC7840 was effective in a slowly progressing 12-month α-synuclein model, even when administered after symptom onset, 4 months post-α-synuclein injection.

These findings highlight the utility of PET/MRI as a non-invasive tool to evaluate drug efficacy and support MCC7840, and other brain-penetrant NLRP3 inhibitors, as promising disease-modifying therapies for Parkinson’s disease, warranting future clinical investigation.

## Teaser

Albornoz *et al.* show using advanced imaging techniques that MCC7840—a novel NLRP3 inflammasome inhibitor—reduces neuroinflammation and protects dopaminergic neurons in preclinical models of Parkinson’s disease. The findings support the investigation of MCC7840 as a potential disease-modifying therapy.

## Introduction

Parkinson’s disease is one of the fastest growing neurodegenerative diseases, with its prevalence projected to reach 12–17 million people by 2040,^1^ primarily driven by an ageing population. Current therapies, such as levodopa (L-DOPA) and deep brain stimulation, can alleviate symptoms but have minimal impact on the underlying disease pathology. Consequently, there is an urgent need for disease-modifying strategies that can halt or slow disease progression.^1^ An emerging consensus suggests that chronic microglia-induced neuroinflammation drives disease progression in Parkinson’s disease.^2,3^ Inflammasomes are multi-protein signalling complexes that function as intracellular sensors of environmental and cellular stress. The NLRP3 (nucleotide-binding domain, leucine-rich repeat, and pyrin domain containing 3) inflammasome is the most well characterized, and its activation involves the oligomerization of NLRP3, recruitment of the inflammasome adapter protein, apoptosis-associated speck-like protein containing a caspase recruitment domain (ASC), and dimerization-induced activation of the caspase-1 protease. Caspase-1 activity results in the cleavage and release of active interleukin (IL)-1β and IL-18, as well as pyroptotic cell death through the cleavage-induced activation of Gasdermin D.^4^ NLRP3 inflammasome activity has been implicated in numerous neuroinflammatory diseases, including Parkinson’s disease.^5-10^ In the Parkinson’s disease brain, the NLRP3 inflammasome can be activated by oxidative stress and by insoluble aggregates of α-synuclein, a major component of Lewy bodies.^8^ Further, a wealth of evidence from clinical studies and preclinical models of Parkinson’s disease have demonstrated pathogenic roles for both caspase-1 and IL-1β in Parkinson’s disease.^6-9,11^

In 2001, Pfizer identified a class of glyburide-inspired diarylsulfonylurea compounds, termed Cytokine Release Inhibitory Drugs (CRIDs), which inhibited IL-1β release.^12^ Initially thought to target glutathione S-transferase omega-1,^13^ it was later discovered in 2015 that CRID3 (CP-456,773; renamed MCC950) specifically inhibits NLRP3 inflammasome activation by binding its NACHT domain (nucleotide-binding and oligomerization domain that mediates ATP-dependent conformational changes) and blocking ATP hydrolysis, thereby preventing inflammasome assembly.^14,15^ This mechanism was recently confirmed by resolving the cryo-electron microscopy structures of NLRP3 bound to MCC950, revealing that its central sulfonylurea group interacts with the Walker A motif of the NLRP3 nucleotide-binding domain and is sandwiched between two arginine residues, explaining the specificity of NLRP3 for MCC950.^16^

In 2018, we demonstrated for the first time that NLRP3 is a viable therapeutic target for Parkinson’s disease.^17^ MCC950 potently inhibited fibrillar α-synuclein-induced inflammasome activation in microglia *in vitro* and attenuated motor decline and dopaminergic neurodegeneration in 6-hydroxydopamine (6-OHDA) and pre-formed α-synuclein fibril (PFF-Syn) mouse models of Parkinson’s disease. We recently replicated these findings, demonstrating that both prophylactic and therapeutic MCC950 administration were effective in these models.^18^ Several subsequent studies have reinforced the role of NLRP3 activation in Parkinson’s disease pathogenesis,^19-23^ and translationally, NLRP3 activation has been observed in human induced pluripotent stem cell (iPSC)-derived microglia and monocytes from Parkinson’s disease patients, correlating with disease severity.^24-28^

Although MCC950 has been widely used as a research tool to confirm NLRP3’s role in over 100 models of human disease,^29^ it is unsuitable for clinical development due to poor pharmacokinetics and other safety concerns.^30^ To overcome these limitations, a series of potent analogues with enhanced pharmacokinetics, brain permeability and target engagement were developed.^28^ Among them, MCC7840 (later named Inzomelid and Emlenoflast by Inflazome and Roche, respectively) was selected as the lead candidate for further investigation based on its superior pharmacokinetics, target engagement and preclinical safety properties.^28^

To evaluate MCC7840’s therapeutic efficacy, we first assessed its pharmacokinetics and metabolism, before testing it in a murine cryopyrin-associated periodic syndrome model of Muckle–Wells syndrome (MWS), which served as a benchmark for NLRP3 inhibition. MWS is a rare autoinflammatory disorder typically driven by mutations in NLRP3 leading to constitutive inflammasome activation, and thus an ideal model to validate NLRP3 inhibitor efficacy. We then tested MCC7840 in two Parkinson’s disease models (6-OHDA and PFF-Syn), using PET/MRI imaging to assess drug efficacy non-invasively in living animals.

A major limitation in translating neuroinflammation-targeting therapies into clinical trials is the lack of sensitive, non-invasive biomarkers that reliably track disease progression and therapeutic response. PET combined with MRI (PET/MRI) is a powerful tool for bridging this translational gap. PET radiotracers such as the translocator protein (TSPO) ligand ^18^F-DPA-714 (for neuroinflammation), fluorodopa (^18^F-FDOPA; for dopamine synthesis) and the dopamine transporter (DAT) ligand ^18^F-FBCTT (for DAT availability) enable longitudinal, whole-brain assessment of neuroinflammatory responses and dopaminergic neuron integrity, both key hallmarks of Parkinson’s disease pathology.

In this study, we utilized these PET radiotracers within the same animal to demonstrate that MCC7840 not only attenuates NLRP3-driven neuroinflammation but also preserves dopaminergic function *in vivo*. These findings highlight PET/MRI as a valuable clinical imaging tool for assessing target engagement and treatment response in future Parkinson’s disease trials. The ability to track neuroinflammation and dopaminergic function in real-time using PET imaging could improve patient stratification, treatment monitoring, and early-phase drug intervention for Parkinson’s disease and other neurodegenerative diseases. By leveraging clinically relevant PET biomarkers, our study establishes a direct translational pathway for MCC7840 from preclinical models to human trials, reinforcing its potential as a disease-modifying therapy for Parkinson’s disease.

## Materials and methods

### Synthesis of MCC950 and MCC7840

MCC950 and MCC7840 were synthesized by coupling of a sulfonamide with an isocyanate under basic conditions. Full synthesis methods have been described previously.^17,28^

### Human monocytic cell line

The human acute monocytic leukemia cell line, THP-1, was cultured in Roswell Park Memorial Institute (RPMI)-1640 medium with 10% fetal bovine serum (FBS) and 1% penicillin-streptomycin. Differentiation into macrophage-like cells was induced with 0.5 μM phorbol 12-myristate-13-acetate (PMA) (Sigma) for 3 h.

### Human monocyte-derived macrophages and microglia

Monocytes were isolated from buffy coats [Australian Red Cross Lifeblood; The University of Queensland (UQ) Human Research Ethics Committee approval]. Peripheral blood mononuclear cells (PBMCs) were separated using SepMate, and CD14+ cells purified with microbeads (Miltenyi).^31^ Human monocyte-derived macrophages (HMDMs) were generated as described.^32^ Monocyte-derived microglia (MDMi) were generated from CD14+ monocytes under serum-free conditions with macrophage-colony stimulating factor (M-CSF), granulocyte-macrophage colony-stimulating factor (GM-CSF), nerve growth factor (NGF)-β, monocyte chemoattractant protein (MCP)-1 and IL-34 for up to 14 days; differentiation was validated by immunofluorescence.^25^

### Primary mouse microglia

Microglia were isolated from postnatal age 0–1 (P0–P1) C57BL/6J mouse brains and cultured in Dulbecco's Modified Eagle Medium (DMEM)-F12 with supplements using a column-free magnetic separation method as previously described.^33^

### NLRP3 inhibitory potency

Cells (1 × 10^5^) were plated in 96-well plates, primed with ultrapure lipopolysaccharide (LPS) (200 ng/ml, 3 h), washed and pre-treated with MCC950 or MCC7840, prior to ATP (5 mM, 1 h). Supernatants were stored for IL-1β ELISA (R&D). Alternate inflammasomes activation and binding studies were performed as described^14^ and extended assay protocols are available in the Supplementary material.

### Animal ethics

Animal procedures were approved by the University of Queensland Animal Ethics Committee and conducted in accordance with the Australian National Health and Medical Research Council guidelines. For studies performed by contract research organizations (CROs), appropriate ethical approvals were obtained from their respective institutional committees.

### Pharmacokinetics

Plasma and brain concentrations of MCC7840 and MCC950 after intravenous (i.v.) or oral dosing in C57BL/6J mice were determined by liquid chromatography-tandem mass spectrometry (LC-MS/MS) as described,^14^ with extended assay protocols provided in the Supplementary material. Additional pharmacokinetic studies including assessments in other species were performed by CROs (methods on request).

### NLRP3 mutant mice (MWS model)

Nlrp3^A350VneoR^ mice were crossed with LysMcre mice. Offspring (Nlrp3^A350V/+LysMcre+^) were injected intraperitoneally (i.p.) with vehicle, MCC950 or MCC7840 every second day from postnatal age 4 (P4) as described,^14^ with studies performed at UQ or by CROs. Body weight and survival were monitored until the end of the experiment at P22.

### 6-OHDA preparation

6-hydroxydopamine (6-OHDA; Sigma) was dissolved in saline with 0.2% ascorbic acid to a final injected dose of 12 μg as previously described.^18^

### Preparation of fibrillar α-synuclein

Recombinant human α-synuclein (rPeptide) was incubated in PBS at 37°C, 400 rpm, for 7 days with daily sonication cycle. Fibril formation was validated by transmission electron microscopy (TEM) and Thioflavin T fluorescence.^34,35^

### Stereotaxic surgery

C57BL/6J mice were anaesthetized with ketamine (100 mg/kg) and xylazine (10 mg/kg), placed in a stereotactic frame, and injected with 2 μl PBS/saline, 6-OHDA (12 μg) or α-synuclein PFFs (8 μg) into the right dorsal striatum at coordinates (anterior-posterior +0.5, medial-lateral −2.0, dorsal-ventral −3.0 mm) as we previously described.^18^ Infusions were delivered at 0.5 μl/min using a 5 μl Hamilton syringe, and the needle was left in place for 5 min post-injection. Mice received analgesics and fluids and were placed on a heat pad until recovery. Extended protocols are provided in the Supplementary material.

### MCC7840 treatment

In 6-OHDA models, mice were dosed orally with MCC7840 daily, starting 24 h pre-surgery until study termination. In PFF models, MCC7840 was delivered in drinking water either prophylactically (1 day before injection) or therapeutically (4 months post-PFF). The Supplementary material contains detailed treatment regimens.

### Plasma IL-1β

Blood was collected in ethylenediaminetetraacetic acid (EDTA) tubes, plasma aliquoted and stored at −80°C. IL-1β was quantified by ELISA (R&D Systems). Extended methods are in the Supplementary material.

### Behavioural tests

All behavioural assays were performed in the light phase. Detailed protocols including acclimatization, and training are in the Supplementary material.

#### Amphetamine induced rotations

D-amphetamine (2 mg/kg) was administered either 17 or 21 days post-6-OHDA; net ipsilateral turns over 10 min were recorded.

#### Balance beam

Mice traversed a 0.5 cm × 1 m beam apparatus; latency to cross was measured at 4, 6, 8 and 10 months post-PFF-Syn.

#### Rotarod test

Accelerated rotarod (5–40 rpm over 5 min) assessed latency to fall at 4, 6, 8 and 10 months post-PFF-Syn as previously described.^17,18^

### Radiotracers

^18^F-DPA-714, ^18^F-FBCTT and ^18^F-FDOPA were synthesized as previously described,^36-38^ or by supplier (ABX). Full synthesis protocols are in the Supplementary material.

### PET/MRI imaging (6-OHDA model)

On Days 24–28 post-surgery, mice underwent PET/MRI with ^18^F-DPA-714 (Day 24), ^18^F-FBCTT (Day 26) and ^18^F-FDOPA (Day 28) to assess microglial activation, dopamine transporter function and presynaptic dopamine synthesis, respectively. Standardized uptake value ratios (SUVRs) were calculated as ipsilateral/contralateral striatum ratios. Detailed acquisition and analysis methods are provided in the Supplementary material.

### PET/MRI imaging (PFF-Syn model)

At 11 months post-PFF injection, mice were scanned with ^18^F-DPA-714 PET/MRI. Regions of interest (ROIs) were anatomically defined in striatum using MRI guidance. SUVRs and T1 relaxation times were calculated. Extended imaging parameters are in the Supplementary material.

### *Ex vivo* autoradiography and immunofluorescence

Mice were injected with ^18^F-DPA-714 (25.4 ± 6.96 MBq) via tail vein, sacrificed 45 min post-injection and brains snap-frozen. Coronal sections (20 μm) were processed for autoradiography; PK11195 (1 mg/kg) confirmed tracer specificity. Adjacent sections were stained for ionized calcium-binding adaptor molecule 1 (Iba1) and glial fibrillary acidic protein (GFAP).^25^ Full staining and autoradiography conditions are detailed in the Supplementary material.

### LC-MS/MS dopamine quantification

Striatal dopamine and metabolites were quantified by LC-MS/MS as previously described.^36^ Full methods are available in the Supplementary material.

### Statistical analysis

All non-imaging data are expressed as means ± standard error of the mean or as box and whisker plots with median (line), 25th to 75th percentiles (box) and full range (whiskers) shown. Statistical analysis was performed with GraphPad Prism software (version 10.2.3). Two-tailed Student’s *t*-test, or one-way analysis of variance (ANOVA) followed by a Dunnett’s or Tukey’s multiple comparison test, was used for comparison of treatment groups to control. A *P*-value <0.05 was considered statistically significant. Extended statistical parameters for all analyses are provided in Supplementary Table 5.

## Results

### MCC7840 is a potent NLRP3 inhibitor with an improved metabolic profile compared with MCC950

**Figure 1:**
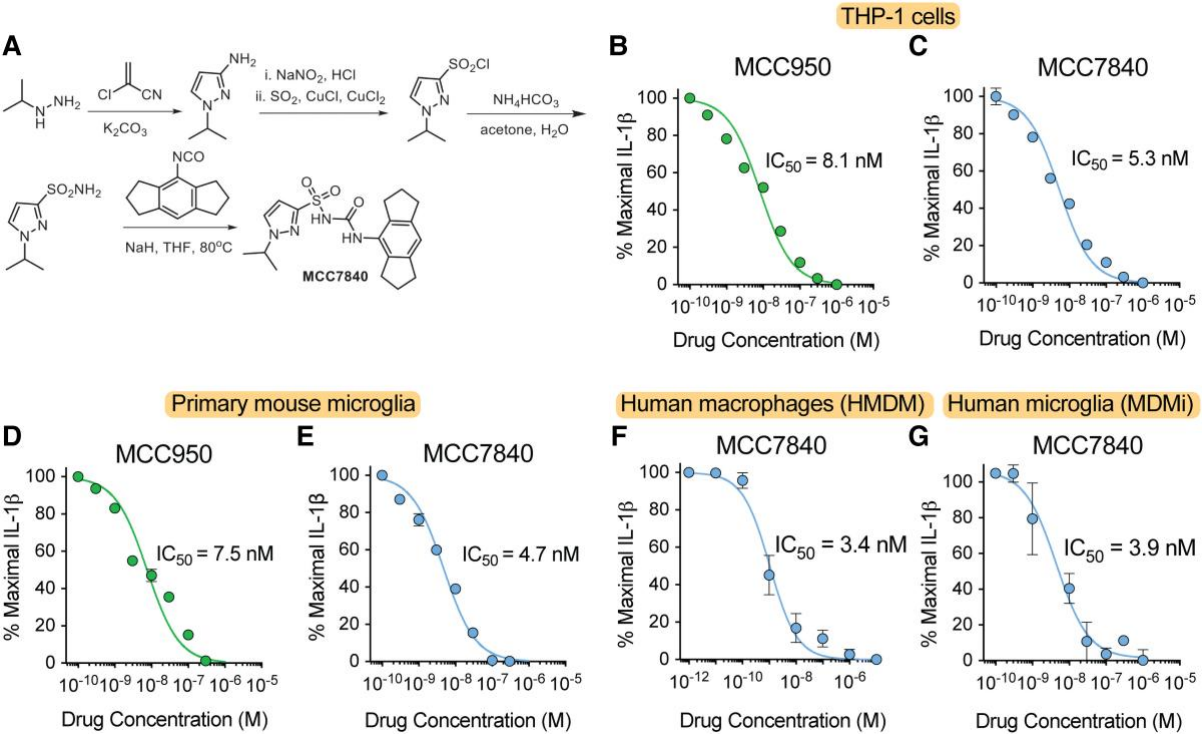
**MCC7840 potently inhibits NOD-leucine rich repeat and pyrin containing protein 3 (NLRP3) activation in human and mouse cells**. (**A**) Overview of the synthetic method for MCC7840 production. (**B**–**G**) MCC950 and MCC7840 dose-dependent inhibition of ATP (5 mM)-induced interleukin (IL)-1β production in: lipopolysaccharide (LPS; 200 ng/ml)-primed human monocytic THP-1 cells (**B** and **C**; *n* = 3); primary mouse microglia (**D** and **E**; *n* = 3); human monocyte-derived macrophages (HMDM) (**F**; *n* = 2 independent donors); and human monocyte-derived microglia (MDMi) (**G**; *n* = 2 independent donors). Data represent mean ± standard error of the mean, with half-maximal inhibitory concentration (IC_50_) values shown.

To identify a drug candidate suitable for clinical development, we developed a series of analogues based on the known sulfonylurea MCC950.^28^ These new sulfonylurea compounds were generally synthesized by coupling a sulfonamide with an isocyanate under basic conditions. Based on initial screening studies and compound physiochemical properties,^17^ MCC7840 was selected for further preclinical evaluation (Supplementary Table 1). The synthesis of MCC7840 required 1-isopropyl-1H-pyrazole-3-sulfonamide, which was prepared in four steps from isopropyl hydrazine hydrochloride and 2-chloroacrylonitrile (Fig. 1A). The hexahydroindacene isocyanate, required for the final synthetic step, was synthesized using established protocols, but since has become commercially available. To compare the NLRP3 inhibitory potency of MCC7840 with MCC950, we utilized LPS-primed human THP-1 macrophages stimulated with ATP to induce inflammasome activation. By measuring IL-1β secretion we demonstrated that MCC7840 could ablate NLRP3 activation with equivalent potency to MCC950 (Fig. 1B and C). Given our focus on central nervous system (CNS) disease, we also assessed activity in primary mouse microglia similarly activated by LPS/ATP to induce inflammasome activation (Fig. 1D and E). MCC7840 exhibited an IL-1β inhibitory potency (IC_50_) of 4.7 nM, consistent with the findings in human myeloid cells. We further confirmed MCC7840’s activity in human monocyte-derived, primary macrophages (HMDM) and primary microglia (MDMi),^25^ both exposed to LPS/ATP, identifying similar IC_50_ values of 3.4 and 3.9 nM (Fig. 1F and G). Finally, we demonstrated that MCC7840 and MCC950 interact at the same binding site on NLRP3, through the use of a radiolabelled competition assay where MCC950 dose-dependently displaced ^3^H-MCC7840 binding to HEK cell membranes expressing human NLRP3 (Supplementary Fig. 1).

Off-target screening confirmed that MCC7840 was inactive at closely related inflammasomes (Supplementary Fig. 2) and was devoid of activity in a broad panel screen of binding, enzyme, kinase and cell-based assays (Supplementary Fig. 3). Comparative *in vitro* metabolism and toxicology studies revealed similar profiles for MCC7840 and MCC950, with the notable exception of cardiac safety (Supplementary Table 2), where MCC7840 exhibited lower human ether-a-go-go-related gene (hERG) ion channel inhibition, suggesting a reduced risk of cardiotoxicity (Supplementary Table 2).

### MCC7840 has an improved pharmacokinetic profile and CNS penetration compared with MCC950

**Figure 2:**
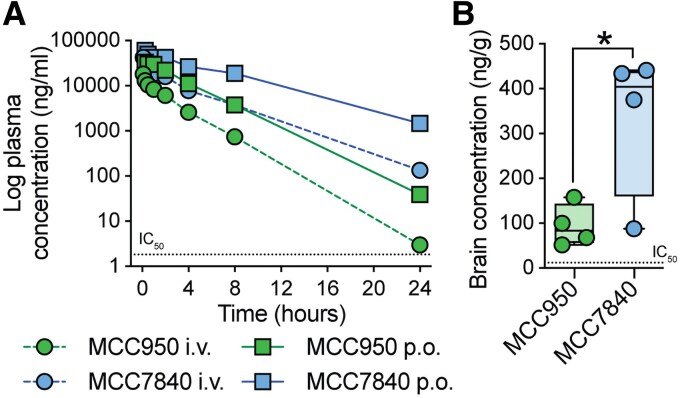
**Oral MCC7840 dosing has improved pharmacokinetics compared with MCC950**. (**A**) 24-h plasma pharmacokinetics of MCC950 and MCC7840 in mice following intravenous (i.v., 4 mg/kg) and oral (p.o., 20 mg/kg) dosing (*n* = 4 mice/group; means ± standard error of the mean shown). Oral half-life, bioavailability: MCC950 2.4 h, 71%; MCC7840 5.0 h, 68%. (**B**) Brain concentrations of MCC950 and MCC7840 2 h following oral dosing (20 mg/kg; *n* = 4/group; box-whisker plot with median (line), 25th to 75th percentiles (box) and full range (whiskers) shown). *P < 0.05 Student’s two-tailed *t*-test. Cell-based half-maximal inhibitory concentration (IC_50_) values for MCC7840 shown with a dotted line.

Single-dose pharmacokinetic studies were next performed with fasted male C57BL/6J mice following intravenous (4 mg/kg) or oral (20 mg/kg) dosing. MCC950 and MC7840 were both well tolerated. However, MCC7840 demonstrated superior pharmacokinetic properties, including higher maximal concentrations (C_max_), area under the curve (AUC) and half-life compared with MCC950 (Fig. 2A and Supplementary Table 3). Low clearance and volume of distribution for MCC7840 were attributed to high plasma protein binding (>99%; Supplementary Table 3). Following oral administration, MCC7840 also achieved higher total brain concentrations than MCC950 (Fig. 2B), with a plasma-to-brain ratio of ∼3.5%. Importantly, both plasma and brain concentrations of MCC7840 remained well above the IC_50_ for NLRP3 inhibition (Fig. 2A and B), supporting oral dosing as a viable route for systemic and CNS NLRP3 inhibition in mice. Oral pharmacokinetics were also assessed in multiple other species (rat, dog, pig, monkey), demonstrating properties consistent with progression to human studies (Supplementary Table 4).

### MCC7840 is highly potent *in vivo*, blocking autoinflammation-mediated mortality in NLRP3 gain-of-function mice

**Figure 3:**
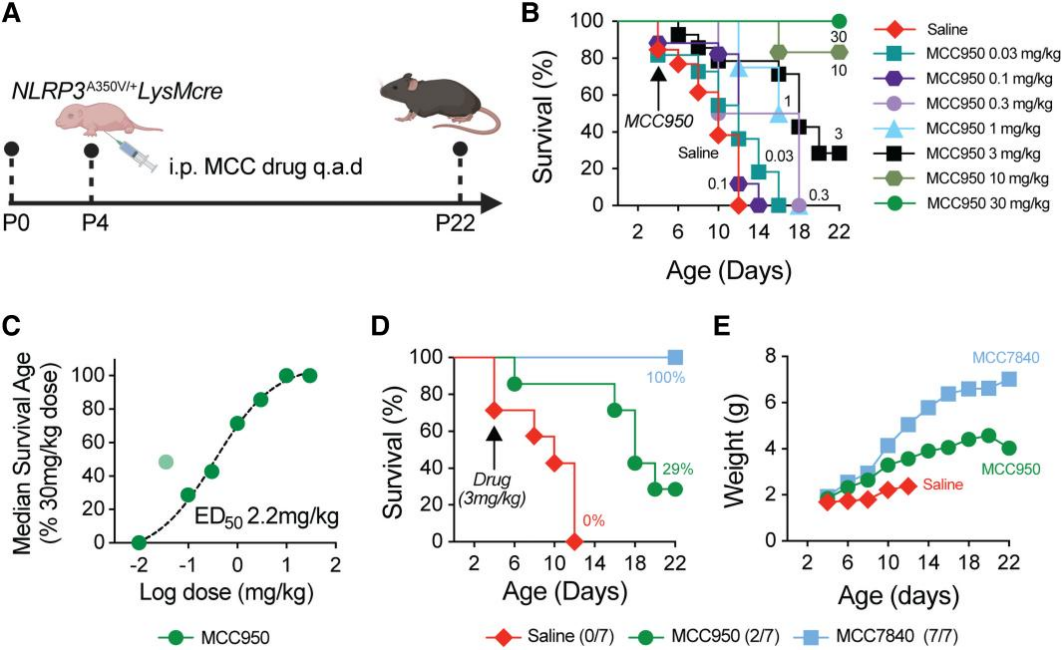
**MCC7840 is highly efficacious and more potent than MCC950 in NOD-leucine rich repeat and pyrin containing protein 3 (NLRP3) gain-of-function mice**. (**A**) Schematic of the NLRP3 gain-of-function *in vivo* studies [created in BioRender. Woodruff, T. (2025) https://BioRender.com/jed3jre]. (**B**) Survival of *Nlrp3*^A350V/+^LysMcre mice treated with increasing doses of MCC950 [0.03–30 mg/kg q.a.d. (every other day)] over 22 days. (**C**) Effective dose (ED) relationship of MCC950 plotted using median survival from **K** to determine an ED_50_ (curve drawn with the 0.1 mg/kg dose excluded). (**D** and **E**) Comparison of survival (**D**) and body weights (**E**) of *Nlrp3*^A350V/+^LysMcre mice administered saline, or MCC950 or MCC7840 dosed at 3 mg/kg q.a.d.

We previously demonstrated that MCC950 is effective in a transgenic mouse model carrying the A350V mutation in NLRP3, that mirrors the pathogenic A352V mutation observed in human patients with the MWS sub-phenotype of cryopyrin-associated periodic syndrome. In this model (i.e. *Nlrp3*^A350V/+^LysMcre mice), NLRP3 is constitutively activated in myeloid cells leading to early-onset systemic inflammation and mortality.^14^ We therefore utilized this model to compare MCC7840 with MCC950. Given that MCC950, when administered at sufficiently high doses, can completely prevent mortality in this model,^14^ we first sought to establish a dose–response relationship to identify an effective dose (ED)_50_ suitable for comparing the efficacy of MCC7840. *Nlrp3*^A350V/+^LysMcre mice were orally dosed with different concentrations of MCC950 (0.03–30 mg/kg) every 2 days from postnatal age 4 (P4) (Fig. 3A), and survival monitored (Fig. 3B). As expected, untreated (saline-gavaged) mice rapidly succumbed to disease, while a 30 mg/kg MCC950 dose fully prevented mortality (Fig. 3B), consistent with our previous findings.^14^ MCC950 doses at 1 mg/kg or lower resulted in all mice succumbing to pathology before model end point (22 days). Plotting the median age of survival across doses revealed an estimated ED_50_ of 2.2 mg/kg (Fig. 3C). We therefore selected 3 mg/kg dosing for future comparative studies. At this dose, MCC7840 provided complete protection from mortality in *Nlrp3*^A350V/+^LysMcre mice, whereas MCC950-treated mice showed significantly reduced survival (Fig. 3D). Body weight trajectories mirrored the survival outcomes (Fig. 3E). These findings demonstrate that MCC7840 is a more potent and efficacious NLRP3 inhibitor *in vivo*, supporting its further development as a therapeutic candidate.

### Oral MCC7840 protects against dopaminergic degeneration in the 6-OHDA Parkinson’s mouse model

**Figure 4:**
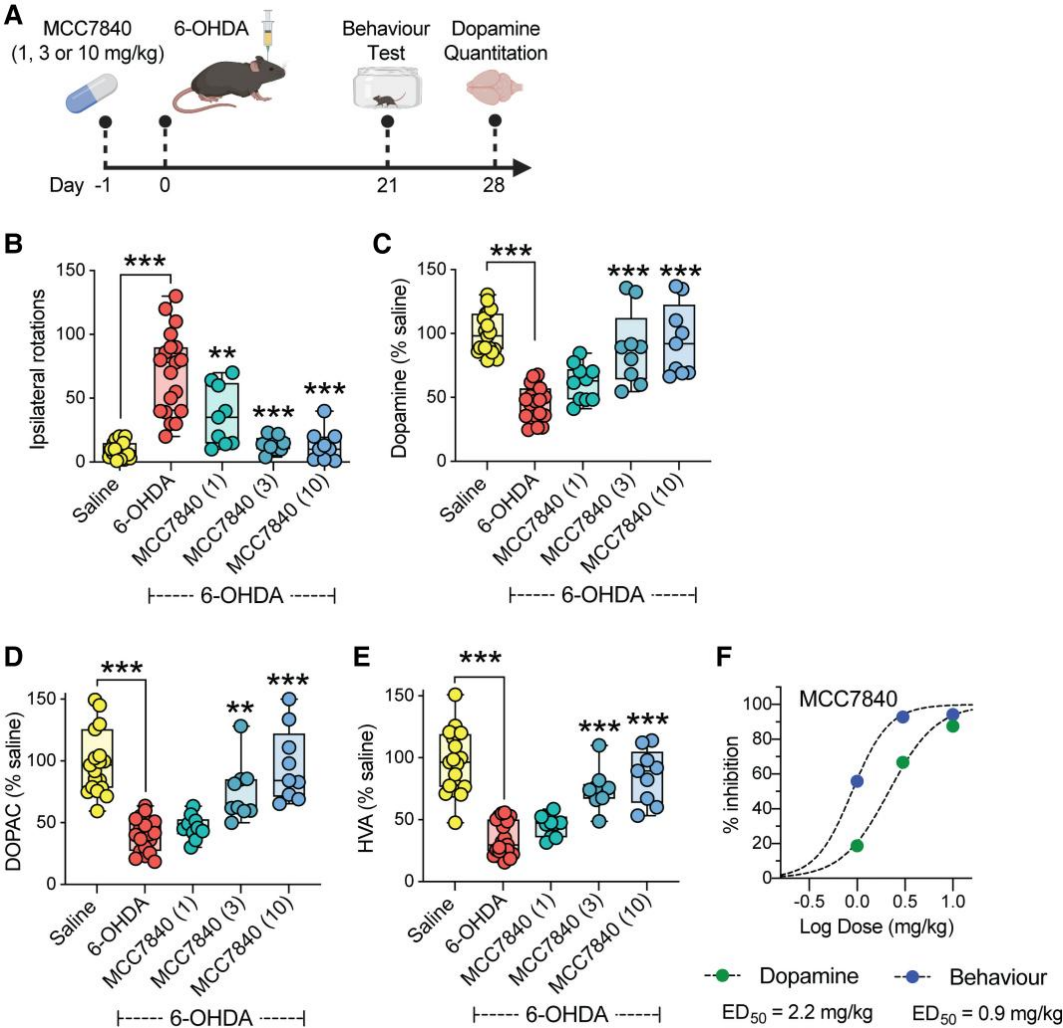
**Oral MCC7840 provides dose-dependent protection in the 6-hydroxydopamine (6-OHDA) model of Parkinson’s disease**. (**A**) Schematic of the 6-OHDA Parkinson’s model [created in BioRender. Woodruff, T. (2025) https://BioRender.com/jed3jre]; mice were injected with 6-OHDA (12 mg) or saline into the striatum on Day 0 (*n* = 19/group). Separate groups of mice (*n* = 9–10/group) were dosed with MCC7840 [1–10 mg/kg oral gavage (p.o.), daily] 1 day prior to 6-OHDA injection. (**B**) Amphetamine-induced ipsilateral rotations at Day 21, and (**C**–**E**) levels of striatal dopamine, 3,4-dihydroxyphenylacetic acid (DOPAC) and homovanillic acid (HVA) 28 days following saline or 6-OHDA injection. (**F**) Effective dose (ED) relationship of MCC7840 plotted using mean ipsilateral rotations and dopamine content from **B** and **C** to determine ED_50_ values for motor behaviour and dopamine loss. Data in **B**–**E** are presented as box-whisker plot with median (line), 25th to 75th percentiles (box) and full range (whiskers) shown. **P < 0.01, ***P < 0.001 by one-way ANOVA (two-tailed), and Dunnett’s post-test comparing to untreated 6-OHDA group.

The unilateral 6-OHDA lesion model induces targeted striatal damage with retrograde degeneration of the nigrostriatal pathway, enabling assessment of both behavioural and neurochemical outcomes relevant to Parkinson’s disease. To evaluate neuroprotective efficacy of MCC7840, mice were orally dosed daily with MCC7840 (1, 3 or 10 mg/kg), starting 1 day before 6-OHDA injection and continuing until study termination (Fig. 4A). In vehicle-treated mice, 6-OHDA lesioning induced a significant increase in amphetamine-induced ipsilateral rotations compared with saline-injected controls (Fig. 4B). MCC7840 treatment at all doses significantly reduced amphetamine-induced rotations in 6-OHDA mice, with maximal efficacy observed at 3 and 10 mg/kg (Fig. 4B). Neurochemical analysis revealed that MCC7840 also protected against striatal dopamine depletion. Quantification of dopamine (Fig. 4C) and its metabolites 3,4-dihydroxyphenylacetic acid (DOPAC) and homovanillic acid (HVA) (Fig. 4D and E) showed significant preservation across all MCC7840 treatment groups. Plotting the average protection across MCC7840 doses in the model revealed an approximate ED_50_ of 0.9 mg/kg for motor behaviour and 2.2 mg/kg for dopamine loss (Fig. 4F).

### MCC7840 ameliorates clinically relevant disease markers in experimental Parkinson’s disease

To further characterize the inhibitory effects of MCC7840 in the 6-OHDA model, we employed PET/MRI imaging to assess markers of both neurodegeneration and neuroinflammation. Imaging of dopamine uptake and dopamine transporter availability is routinely used in human Parkinson’s disease patients to monitor dopaminergic degeneration, and may serve as a valuable biomarker in clinical trials.^39^ Given the upregulation of microglial activity in Parkinson’s disease and the proposed mechanism of action of NLRP3 inhibition,^40^ we also imaged a neuroinflammation biomarker (TSPO).^41^

**Figure 5:**
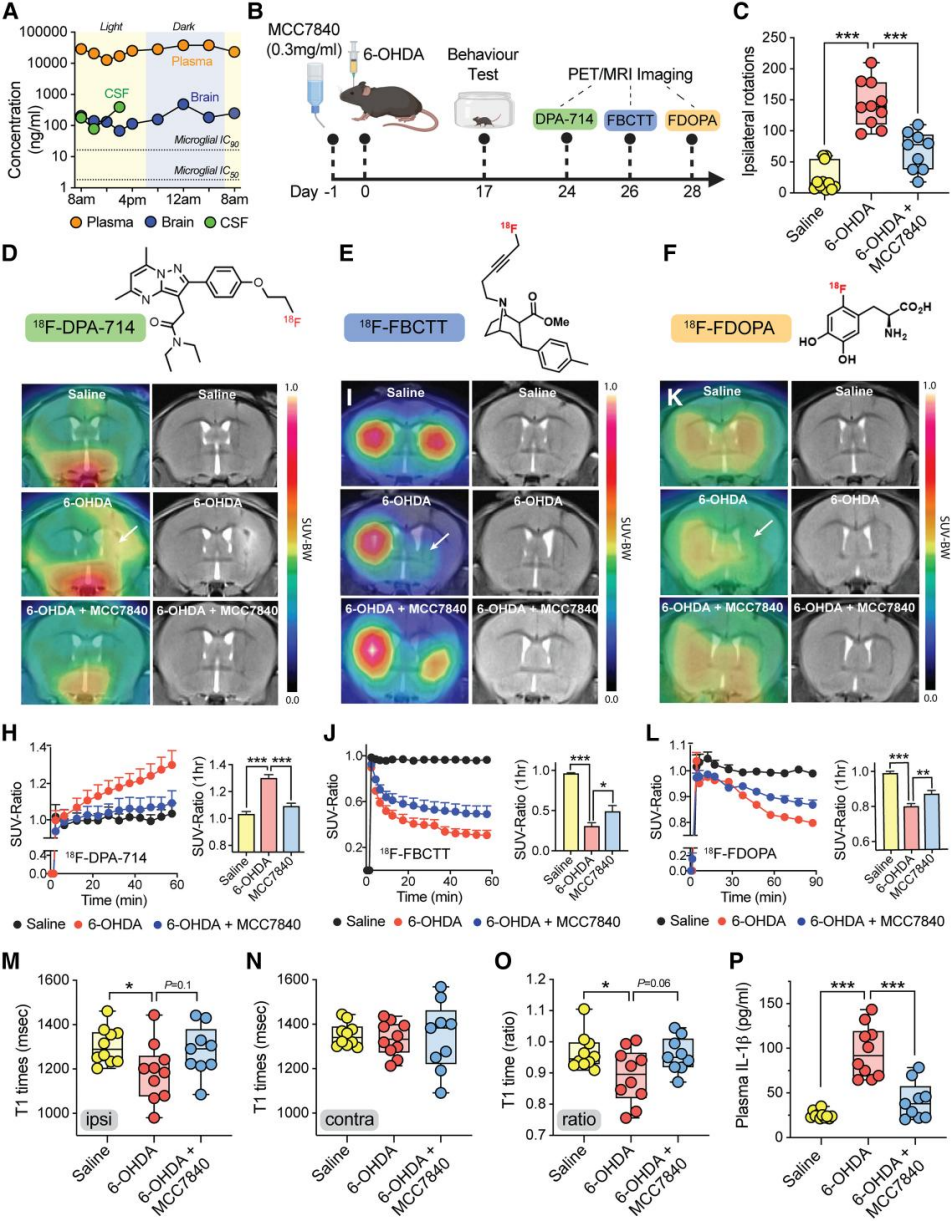
**MCC7840 oral treatment in the 6-OHDA model of Parkinson’s disease decrease *in vivo* PET/MRI imaging markers of neurodegeneration and neuroinflammation**. (**A**) Twenty-four hour pharmacokinetics of MCC7840 administered in drinking water (0.3 mg/ml), with concentrations shown in plasma, CSF and perfused brain. The microglial IC_50_ and IC_90_ values are shown with dotted lines. (**B**) Schematic of the 6-OHDA imaging study [created in BioRender. Woodruff, T. (2025) https://BioRender.com/jed3jre]; mice were injected with 6-OHDA (12 mg; *n* = 10) or saline (*n* = 11) into the striatum on Day 0, followed by motor behaviour testing, and PET imaging for translocator protein ligand ^18^F-DPA-714, dopamine transporter ligand ^18^F-FBCTT, and fluorodopa ^18^F-FDOPA on Days 24, 26 and 28. A group of mice (*n* = 9) were treated with MCC7840 in drinking water (0.3 mg/ml) 1 day prior to 6-OHDA injection. (**C**) Amphetamine-induced ipsilateral rotations at Day 17. (**D**–**F**) Structures of ^18^F-DPA-714, ^18^F-FBCTT and ^18^F-FDOPA. (**G**) Representative brain PET/MRI images of ^18^F-DPA-714 in the study; arrow shows area of increased PET signal in the untreated 6-OHDA mouse striatum. (**H**) Time-activity curves for ^18^F-DPA-714 generated from dynamic imaging and presented as standard uptake value ratio from ipsilateral to contralateral hemispheres (standardized uptake value ratio, SUV-ratios). The 60 min imaging time point is shown on the right. (**I**) Representative brain PET/MRI images and (**J**) quantitative SUV-ratios for ^18^F-FBCTT. The arrow in **I** shows area of PET signal loss in ipsilateral 6-OHDA mouse striatum. (**K**) Representative brain PET/MRI images and (**L**) quantitative SUV-ratios for ^18^F-FDOPA. The arrow in **K** shows area of PET signal loss in ipsilateral 6-OHDA mouse striatum. (**M**–**O**) MRI blood–brain barrier leakage measurement of gadolinium T1 relaxation time values in ipsilateral (**M**) and contralateral (**N**) hemispheres and expressed as a ratio (**O**). (**P**) Plasma IL-1β concentrations in mice at Day 28. Data in **C** and **M**–**P** are presented as box-whisker plot with median (line), 25th to 75th percentiles (box), and full range (whiskers) shown, and in **H**–**L** as means ± standard error of the mean. *P < 0.05, **P < 0.01, ***P < 0.001 by one-way ANOVA (two-tailed), and Dunnett’s post-test comparing to untreated 6-OHDA group. SUV-BW = standard uptake value corrected for body weight.

We opted for drinking water administration of MCC7840 for these experiments based on prior efficacy observed with MCC950 using this route,^17,18^ and to minimize handling stress associated with daily oral gavage, which could confound live imaging outcomes. We first confirmed that MCC7840 achieves sufficient brain exposure via this route. Mice were given free access to MCC7840 (0.3 mg/ml in drinking water) for five consecutive days and then blood and brain samples collected over a 24 h period. MCC7840 achieved consistent steady-state levels in both compartments, with concentrations exceeding those required for NLRP3 inhibition in microglia (Fig. 5A). CSF samples from a separate cohort of MCC7840-dosed mice confirmed that concentrations matched those in brain tissue at the time points investigated (Fig. 5A), supporting sufficient CNS exposure for target engagement during chronic dosing.

Three experimental groups were examined: saline-injected (control), 6-OHDA-injected (disease) and 6-OHDA-injected mice treated with MCC7840 in the drinking water (0.3 mg/ml) (Fig. 5B). To perform PET imaging, the same mouse was intravenously administered three radiotracers at sequential time points: ^18^F-DPA-714 at Day 24 to label TSPO and neuroinflammation (Fig. 5D), ^18^F-FBCTT at Day 26 to label dopamine transporter (DAT) availability (Fig. 5E)^42^ and ^18^F-FDOPA at Day 28 to label dopamine uptake (Fig. 5F),^43^ following 6-OHDA or saline injection. At each time point, mice were anaesthetized and imaged using PET/MRI to visualize radiotracer uptake in the brains of living mice (Fig. 5B).

We confirmed successful lesioning and the efficacy of MCC7840 by measuring amphetamine-induced ipsilateral rotations at Day 17 post-surgery (Fig. 5C). One week after the amphetamine test, we then conducted PET/MRI imaging studies, beginning with ^18^F-DPA-714 to assess neuroinflammation. Increased signal in the ipsilateral hemisphere was observed in 6-OHDA mice compared with both saline controls, and MCC7840-treated 6-OHDA mice (Fig. 5G). Time-activity curves generated from dynamic imaging of ^18^F-DPA-714 revealed a significant increase in the standard uptake value (SUV) ratios between lesioned (ipsilateral) and non-lesioned (contralateral) hemispheres in 6-OHDA mice, which was almost entirely inhibited by MCC7840 treatment (Fig. 5H). Following a 2-day washout period, mice were then injected with ^18^F-FBCTT to image DAT loss. A profound reduction in ipsilateral signal was observed in 6-ODHA mice compared with control mice (Fig. 5I). MCC7840 treatment resulted in a significant, but not complete protection from the loss of signal, as measured by dynamic imaging (Fig. 5J). After another 2-day washout, ^18^F-FDOPA was administered to assess dopamine uptake. Similar to DAT imaging, 6-OHDA mice showed reduced ipsilateral ^18^F-FDOPA signal, which was significantly protected by MCC7840 (Fig. 5K and L).

As we utilized a combined PET/MRI approach, we were also able to investigate blood–brain barrier integrity via gadolinium contrast-enhanced MRI. T1 relaxation times were measured, as leakage of gadolinium into brain tissue shortens T1 values. Quantification of T1-weighted signals in both ipsilateral (Fig. 5M) and contralateral hemispheres (Fig. 5N) demonstrated a significant T1 decrease in untreated 6-OHDA mice, which was absent in MCC7840-treated animals indicating protection against blood–brain barrier disruption (Fig. 5O). Finally, to confirm target engagement, plasma IL-1β levels were measured at study end point, revealing that MCC7840 effectively suppressed 6-OHDA-induced increase in IL-1β, consistent with *in vivo* NLRP3 inhibition (Fig. 5P).

### MCC7840 demonstrates efficacy in the PFF-Syn mouse model under both preventative and therapeutic dosing regimens

**Figure 6:**
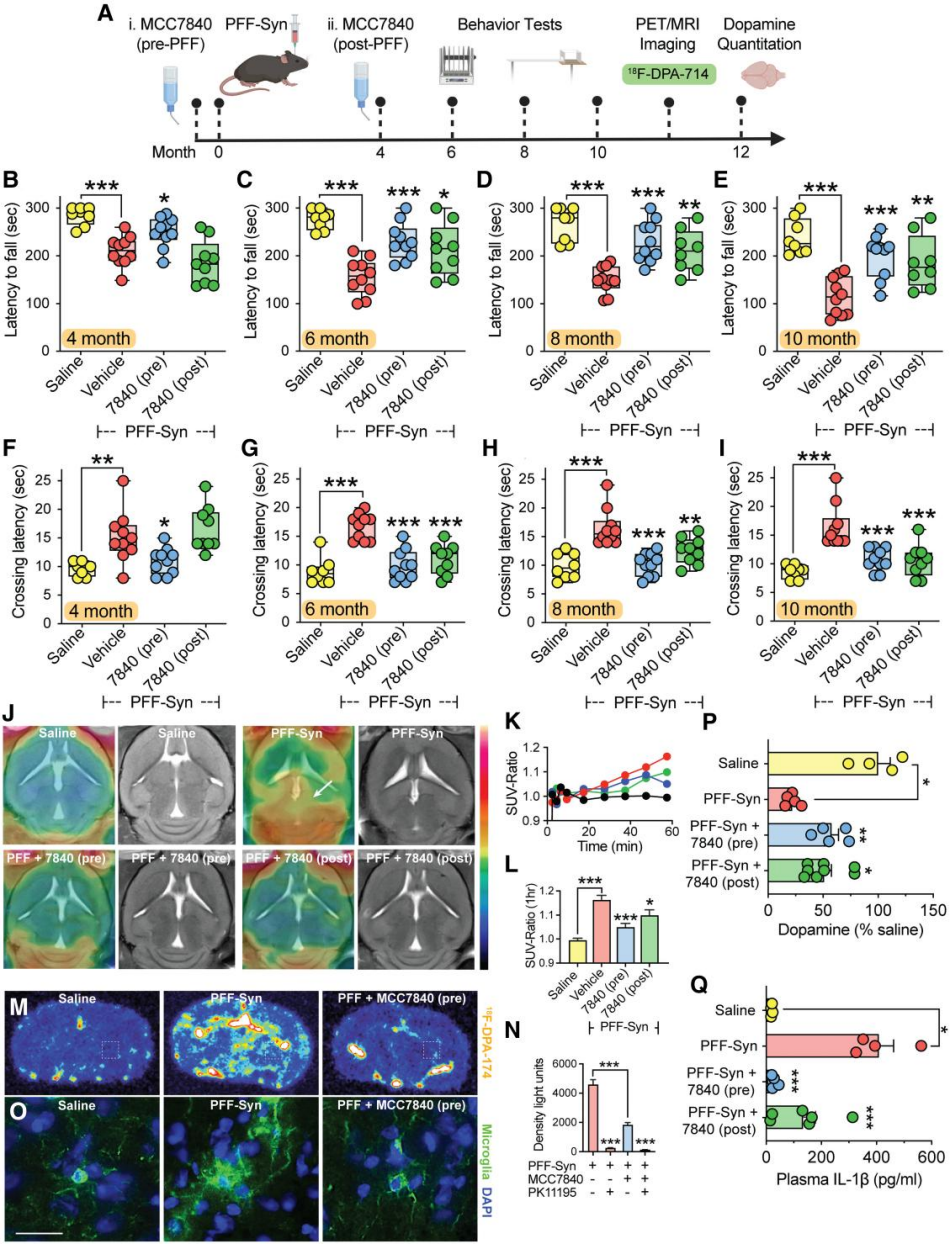
**Prophylactic or delayed oral MCC7840 treatment ameliorates dopamine degeneration and neuroinflammation in the pre-formed α-synuclein fibril (PFF-Syn) Parkinson’s disease mouse model**. (**A**) Schematic of the PFF-Syn study [created in BioRender. Woodruff, T. (2025) https://BioRender.com/jed3jre]; mice were injected with PFF-Syn (8 μg) or saline into the striatum on Day 0, followed by motor behaviour testing from 4 to 10 months, and PET imaging for translocator protein ligand ^18^F-DPA-714 at 11 months. Separate groups of mice were treated with MCC7840 in drinking water (0.3 mg/ml) 1 day prior to PFF-Syn injection (7840 pre) or 4 months after PFF-Syn injection (7840 post). (**B**–**E**) Rotarod and (**F**–**I**) balance-beam testing of mice at 4, 6, 8 and 10 months post PFF-Syn or saline injection (*n* = 8–10/group). (**J**) Representative brain PET/MRI images of ^18^F-DPA-714 in the study; arrow shows area of increased PET signal in the untreated PFF-Syn mouse striatum. (**K**) Time-activity curves for ^18^F-DPA-714 generated from dynamic imaging and presented as standard uptake value ratio (SUV-ratio) from ipsilateral to contralateral hemispheres (*n* = 4–8/group). (**L**) Sixty minute SUV-ratio time point for data in **L**. (**M**) Representative autoradiography images for ^18^F-DPA-714 from brain slices taken following PET imaging. (**N**) Quantitation of ^18^F-DPA-714 autoradiography, and blockade of signal with PK11195. (**O**) Representative immunohistochemistry for microglial marker ionized calcium-binding adaptor molecule 1 (Iba1; green) from brain slices taken following PET imaging; cell nuclei 4′,6-diamidino-2-phenylindole (DAPI; blue). Square dotted line box in **M** represents approximate region imaged; magnification ×40, scale bars = 20 μm. (**P**) Ipsilateral striatal dopamine quantitation, and (**Q**) plasma interleukin (IL)-1β concentrations in mice after completion of PET imaging experiments (*n* = 4–8/group). Data in **B**–**I** represent means ± standard error of the mean, all other data are presented as box-whisker plot with median (line), 25th to 75th percentiles (box) and full range (whiskers) shown. *P < 0.05, **P < 0.01, ***P < 0.001 by one-way ANOVA (two-tailed), and Dunnett’s post-test comparing to untreated PFF-Syn group.

To confirm the efficacy of MCC7840 in a chronic Parkinson’s disease model that replicates the synucleinopathy observed in human patients, we utilized the PFF-Syn model building on our prior demonstration of efficacy with MCC950.^17,18^ We designed a PFF-Syn experiment to compare MCC7840 administration either prior to PFF-Syn injection (preventative, pre-PFF), or after motor symptoms had emerged 4 months post-injection (therapeutic, post-PFF) (Fig. 6A). At the 4-month time point, PFF-Syn-injected mice exhibited significant motor impairment on the rotarod test compared with saline-injected mice. This deficit was significantly attenuated in mice pre-treated with MCC7840 (preventative group), but not in mice that had not yet received MCC7840 (therapeutic group) as expected (Fig. 6B). At 6, 8 and 10 months post-injection, untreated PFF-syn mice showed progressive motor decline, whereas both preventative and therapeutic MCC7840 groups were significantly protected (Fig. 6C–E). Similar results were observed in the balance beam test. At 4 months, untreated PFF-Syn mice showed increased crossing latency, which was significantly reduced in MCC7840 pre-treated mice (Fig. 4F). At later time points (6, 8 and 10 months), both preventative and therapeutic MCC7840 groups showed improved balance beam performance compared with untreated PFF-Syn mice (Fig. 6G–I).

Given the neuroinflammation findings from ^18^F-DPA-714 PET imaging in the 6-OHDA model, we applied the same modality to this PFF-Syn cohort (Fig. 6J). PET/MRI imaging at 12 months revealed elevated ^18^F-DPA-714 signal in PFF-Syn-injected mice compared with saline-injected mice. This signal was significantly reduced in both preventative and therapeutic MCC7840 treated groups (Fig. 6K and L). To further confirm tracer specificity, we dissected brains immediately after PET imaging in a subset of mice, and performed *ex vivo* autoradiography in brain slices in the presence or absence of PK11195, a specific high-affinity ligand to the mitochondrial TSPO binding site (Fig. 6M). This validated the specificity of ^18^F-DPA-714 and confirmed reduced neuroinflammation radiotracer signal in MCC7840-treated mice (Fig. 6N). To further support the use of ^18^F-DPA-714 as a proxy for microglial activation we used the same tissue sections to stain against the microglial marker Iba1, which demonstrated pronounced microgliosis in the ipsilateral site of PFF-Syn-injected mice compared with saline and MCC7840-treated groups (Fig. 6O). Astrocytic activation was also assessed using GFAP immunostaining, demonstrating astrogliosis in the PFF-Syn-injected mice and reduced GFAP staining in PFF-Syn mice treated with MCC7840 (Supplementary Fig. 4).

Assessment of blood–brain barrier integrity using gadolinium-enhanced MRI showed no detectable changes in T1 relaxation times across groups (Supplementary Fig. 5), indicating that blood–brain barrier leakage was not evident at this time point in the PFF-Syn model.

Following PET imaging, mice were euthanized and striatal dopamine and metabolites were quantified. PFF-Syn-injected mice demonstrated significant dopamine loss (Fig. 6P) and reduced metabolite levels (Supplementary Fig. 6), both of which were significantly protected by MCC7840 under both dosing regimens. Finally, plasma IL-1β levels were measured as a peripheral biomarker of inflammasome activation. PFF-Syn injection induced a ∼20-fold increase in IL-1β, which was fully suppressed in the MCC7840 preventative group and reduced by ∼70% in the MCC7840 therapeutic group (Fig. 6Q).

## Discussion

Chronic, low-grade inflammation is now widely recognized as a key driver of ageing^44^ and neurodegeneration.^45^ Studies in humans and animal models show that the expression of damage-associated molecular patterns leading to inflammaging phenotypes are linked to increased morbidity and mortality.^46,47^ The NLRP3 inflammasome has become a focal point in drug discovery due to its central role as a danger sensor and its proposed involvement in age-related diseases, including neurodegenerative disorders.^10,48,49^ While several NLRP3 inhibitors show promise in murine models of acute and chronic inflammation,^50^ none are yet approved for clinical use.^30^ In this study, we designed and evaluated MCC7840 (also referred to as Inzomelid or Emlenoflast) as a potent and selective NLRP3 inflammasome inhibitor. We demonstrated improved oral pharmacokinetics and efficacy of MCC7840 compared with MCC950 in a mouse model of MWS, and validated its efficacy in two mouse models of Parkinson’s disease, focusing on clinically relevant PET/MRI biomarkers^39^ to support potential future clinical studies in Parkinson’s disease patients.

MCC950 contains a 2-methylfuran moiety that was previously flagged as a putative toxicophore.^51^ Oxidative metabolism of the furan can result in the reactive aldehydes such as acetylacrolein and methylbutenedial, which were identified as the principal reactive intermediates of 2- and 3-methylfurans that bind covalently to tissue macromolecules in hepatic and pulmonary microsomal systems.^52^ In MCC7840, this furan motif was replaced with an isopropylpyrazole, resulting in similar *in vitro* potency to MCC950 across human and mouse macrophages and microglia. However, MCC7840 demonstrated improved *in vivo* pharmacokinetic properties, including higher maximal plasma concentrations, and an extended plasma half-life after oral dosing. This improved plasma pharmacokinetic profile correlated with significantly improved efficacy in the MWS mouse model. Furthermore, MCC7840 showed improved brain permeation, with concentrations nearly 4-fold higher than MCC950 2 h after oral dosing in healthy mice. Given our observation of blood–brain barrier leakiness in the 6-OHDA model, it is likely that brain exposure would be further increased when dosed in the Parkinson's disease models, although this hypothesis requires experimental validation. Taken together, these data support MCC7840 as a preclinical candidate with excellent orally bioavailability, *in vivo* efficacy and blood–brain barrier penetration at therapeutically relevant concentrations far superior to MCC950.

Safety is a critical consideration in the clinical translation of therapeutic candidates. MCC950 was previously tested in Phase II clinical trials for rheumatoid arthritis, but was not developed further due to its poor clinical pharmacokinetics and association with adverse events.^53^ By contrast, MCC7840 has demonstrated excellent safety, tolerability and pharmacokinetic profile in Phase I randomized, double-blinded, placebo-controlled, single and multiple ascending dose clinical trials in healthy subjects (NCT04015076), with related analogues also showing safety and tolerability in humans (NCT04086602).

Most preclinical studies of NLRP3 inhibition in models of neurodegeneration have employed prophylactic treatment regimens.^17,21,54^ Our previous work showed that prophylactic administration of MCC950 in the human PFF-Syn model is neuroprotective, preventing motor deficits and TH-positive neuronal loss, associated with reduced phosphorylated (pSer129) α-synuclein accumulation, oxidative stress, and levels of cleaved-caspase 1 and ASC.^17^ Here, we demonstrate that therapeutic intervention with MCC7840, initiated 4 months after PFF-Syn intrastriatal injection, remains efficacious. At this time point, PFF-Syn animals display significant motor dysfunction compared with saline-injected animals. Interestingly, both prophylactic and therapeutic treatments conferred similar neuroprotection, as reflected in the motor behaviour, PET imaging and immunostaining of microglial activation, and levels of dopamine, DOPAC and HVA measured at the end of the 12-month study. These findings suggest that inhibition of microglial NLRP3 inflammasome activity can prevent ongoing dopamine loss even in mice with established synucleinopathy and symptomatic disease.

Parkinson’s disease is defined by the International Parkinson and Movement Disorders Society as ‘a core clinical motor syndrome (Parkinsonism) accompanied by substantia nigra pars compacta neurodegeneration and synuclein deposition’.^55^ However, this description is based on post-mortem studies, and the current lack of reliable biomarkers in living patients poses a major challenge for early diagnosis and for evaluating the efficacy of novel therapeutics in clinical trials. The development of radiotracers combined with PET imaging to assess *in vivo* dopaminergic neuron loss, alongside microglial activation and neuroinflammation, may offer a promising strategy to evaluate anti-inflammatory drugs in the degenerating CNS.

The radiotracer ^18^F-FBCTT is employed for imaging the dopamine transporter, DAT.^38^ In clinical practice, DAT imaging is used to evaluate the functional integrity of striatal dopaminergic neurons and characterize early Parkinson’s disease.^56^ Preclinical models of Parkinson’s disease have previously used this radiotracer to assess the maturation of transplanted dopaminergic neurons in a rat 6-OHDA model.^42^ Our results support the use of ^18^F-FBCTT imaging to assess dopaminergic neuronal loss in the 6-OHDA mouse model, where a dramatic loss of signal was observed in the ipsilateral hemisphere following 6-OHDA injection. Consistent with our motor behaviour findings, MCC7840 protected against the loss of DAT signal, supporting the utility of this probe for evaluating therapeutic neuroprotection *in vivo*.

^18^F-FDOPA PET imaging directly probes nigrostriatal dopamine synthesis,^57^ allowing for the assessment of levodopa (L-DOPA) metabolism. It has historically been used in Parkinson’s disease patients to measure dopaminergic function.^58^ In preclinical models, decreased ^18^F-FDOPA uptake correlates with the severity of dopaminergic dysfunction in unilaterally 6-OHDA-lesioned rats, as demonstrated through comparisons with biochemical, immunohistochemical and behavioural measurements.^43^ Our results are consistent with these findings, showing reduced ^18^F-FDOPA PET uptake at the 6-OHDA injection site. Although the signal change was less robust than observed with ^18^F-FBCTT, MCC7840 treatment protected against ^18^F-FDOPA signal loss, in line with improvements in motor behaviour and dopamine neurotransmitter levels.

In this study, we also used ^18^F-DPA-714, a second-generation selective ligand radiotracer for TSPO, which is predominantly expressed on activated microglia.^37^ This radiotracer has been clinically validated for detecting early microglial activation in Alzheimer’s disease patients,^59^ where TSPO uptake was positively correlated with amyloid-beta deposition.^60^ In Parkinson’s disease patients, increased microglial activation has been demonstrated using this tracer in the nigrostriatal pathway and the frontal cortex.^61^  ^18^F-DPA-714 was also used in preclinical models as an *in vivo* marker of early microglial activation, including in a rat 6-OHDA ^18^F- and a rat model of progressive dopaminergic degeneration using intranigral injections of viral vectors encoding synuclein.^62^ In this study, we corroborated these findings in mice injected with 6-OHDA, showing increased ^18^F-DPA-714 uptake on the ipsilateral side, compared with the contralateral side. Importantly, MCC7840 treatment ameliorated the 6-OHDA-induced ^18^F-DPA-714 signal, indicating that MCC7840 reduces microglial NLRP3-driven neuroinflammation in the brain. While TSPO is a widely used marker of neuroinflammation, its expression is not exclusive to microglia. Future studies could consider more specific PET ligands such as CSF1R to better delineate specific microglial activation.

Here, we also report for the first time the use of ^18^F-DPA-714 to assess *in vivo* microglial activation in the mouse PFF-Syn model.^34^ At 12 months, an increase in radiotracer uptake was observed, indicating a pattern of microglial activation not just at the injection site but also spreading throughout the brain, consistent with the known propagation PFF-Syn pathology in this model.^34,63^ Notably, this marker of neuroinflammation was reduced in both prophylactic and therapeutic settings, suggesting a direct role for the NLRP3 inflammasome in PFF-Syn spreading and microglial activation, thus validating an *in vivo* approach to assess drug efficacy in future clinical trials. We also supported the ^18^F-DPA-714 findings, by performing immunostaining for microglia (Iba1) on brain sections collected after imaging, which confirmed increased microglial activation in PFF-Syn-injected mice and its attenuation by MCC7840 treatment. Dopaminergic PET imaging was not performed in the PFF-Syn model due to practical constraints including animal age, anaesthesia burden and study duration; however, future studies could explore correlations between neuroinflammation and direct dopaminergic imaging markers in this model. We acknowledge that further studies are required to clarify the neuropathological links between microglial NLRP3 activation and dopaminergic cell death, and to extend behavioural assays to more sensitive indicators of nigrostriatal degeneration relevant to Parkinson’s disease.

Taken together, these data provide further support that NLRP3 inflammasome activation plays a key pathological role in the development and progression of Parkinson’s pathology in preclinical models. MCC7840 displays highly potent inhibitory activity on human and mouse microglia, improved metabolic and toxicological properties, CNS pharmacokinetics, pharmacokinetic/pharmacodynamics in a monogenic gain-of-function model of NLRP3 activation (MWS mice), and efficacy in two distinct preclinical models of Parkinson’s disease. Notably, delayed therapeutic intervention with MCC7840 remained efficacious in the PFF-Syn model, aligned with reductions in ^18^F-DPA-714 neuroinflammation signals in living mice. Our results therefore support the clinical testing of NLRP3-targeting drugs such as MCC7840 in Parkinson’s disease and other age-related neurodegenerative disorders.^64^ Beyond Parkinson’s disease, NLRP3 activation has been implicated in Alzheimer’s disease, amyotrophic lateral sclerosis, multiple sclerosis, Huntington’s disease and in subsets of psychiatric conditions with an inflammatory component, suggesting that MCC7840 may have broader therapeutic applications that merit disease investigation.

## Supplementary Material

awaf372_Supplementary_Data

## Data Availability

The data supporting the findings of this work are available within the manuscript and Supplementary material. Should any raw data files be needed in another format, they are available from the corresponding author upon reasonable request.
