# Supplementary material for: PET-MRI biomarkers reveal efficacy of a novel NLRP3 inhibitor in Parkinson’s disease models
Source: Brain. 2025 Oct 16;149(4):1289–301. doi: 10.1093/brain/awaf372 (PMC13058460; doi:10.1093/brain/awaf372)
Supplement: awaf372_Supplementary_Data [file awaf372_supplementary_data.pdf]

Supplementary Figure 1

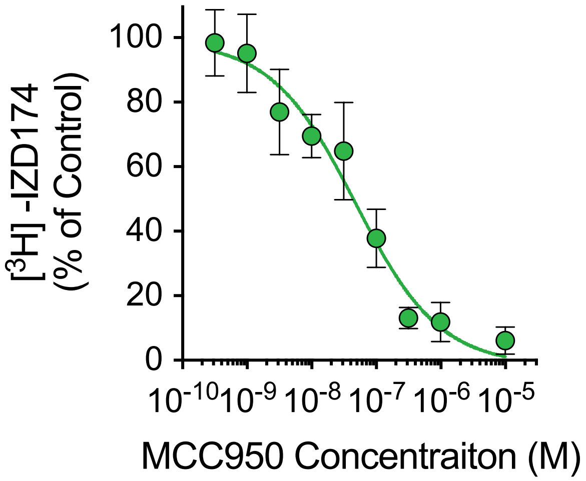

Supplementary Figure 1: MCC7840 and MCC950 bind to the same site on NLRP3. Dose-dependent competition displacement of the radioligand [^{3}H]-MCC7840 by MCC950 in NLRP3 over-expressing HEK293 cell lysates (n=4; MCC950 IC_{50} = 47nM).

Supplementary Figure 2

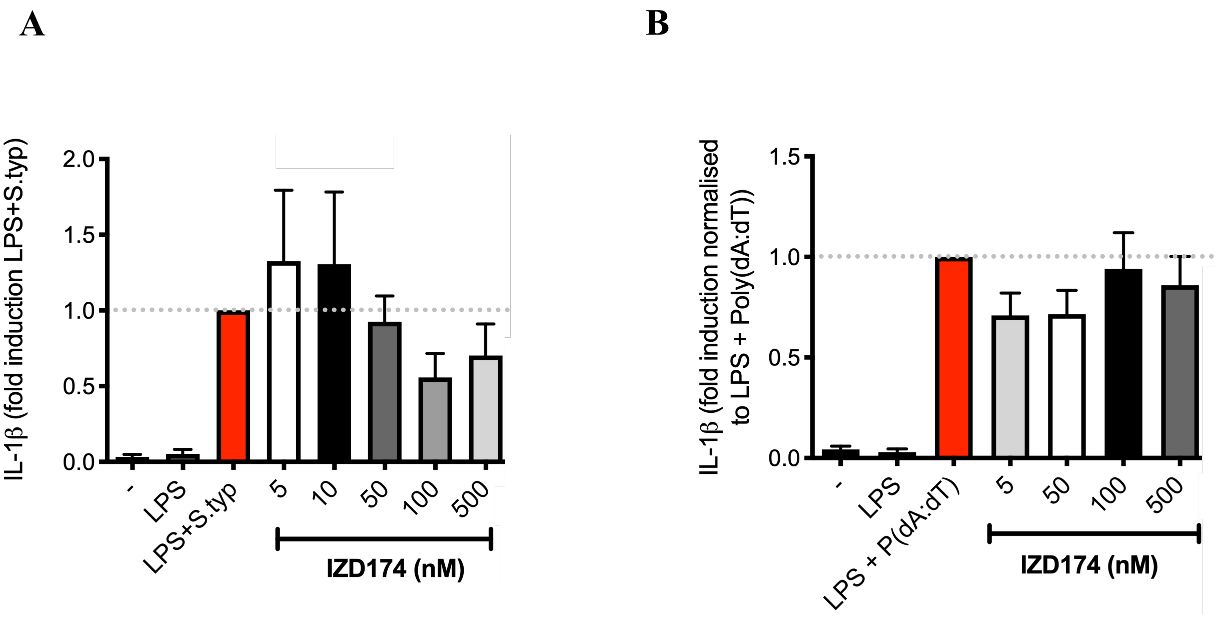

Supplementary Figure 2: MCC7840 is not active at NLRC4 and AIM2 inflammasomes. Bone marrow cells were isolated from C57BL/6J mice and cultured macrophages stimulated with *S. typhimurium* (S.typ) and poly(dA:dT) to activate [**A**] NLRC4 and [**B**] AIM2 inflammasomes. No significant inhibitory activity was observed for MCC7840 at the concentrations tested (n=2; mean ± SEM).

Supplementary Figure 3

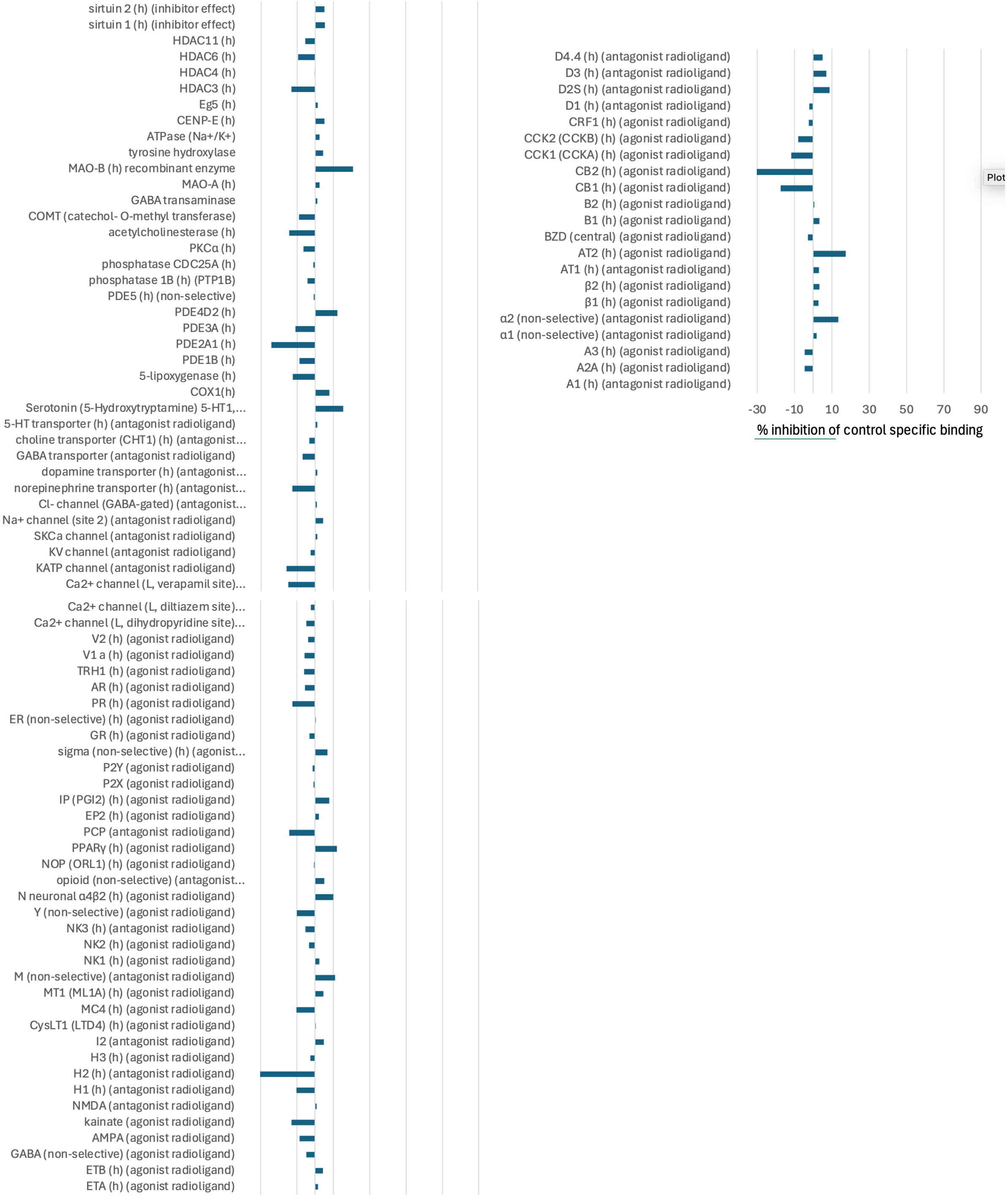

Supplementary Figure 3: MCC7840 does not have significant off-target activities. MCC7840 (10uM) was screened for inhibition or activation activities at 95 targets (Eurofins Cerep panel) in binding and enzyme and uptake assays. No activity at >35% was observed for any of the targets.

Supplementary Figure 4

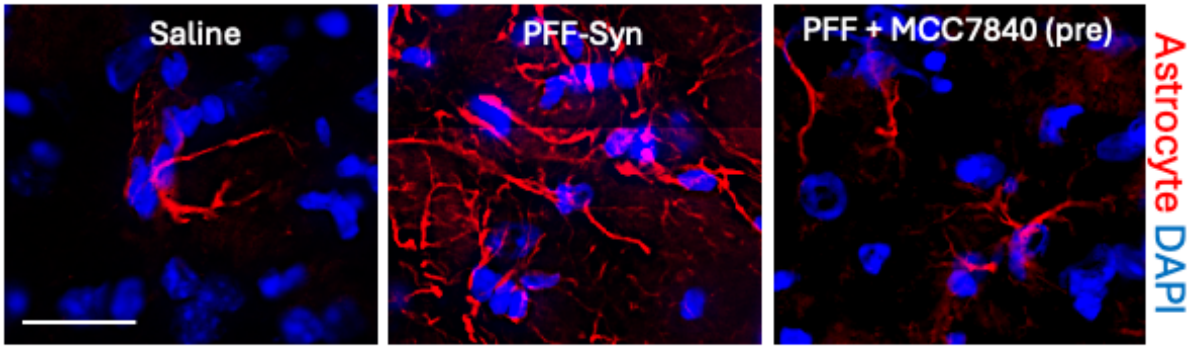

Supplementary Figure 4. Prophylactic or delayed oral MCC7840 treatment ameliorates neuroinflammation in the PFF-Syn Parkinson's disease mouse model. Representative immunohistochemistry for astrocyte marker GFAP (red) from brain slices taken following PET imaging; cell nuclei DAPI (blue). Magnification, ×40. Scale bars, 20 µm.

Supplementary Figure 5

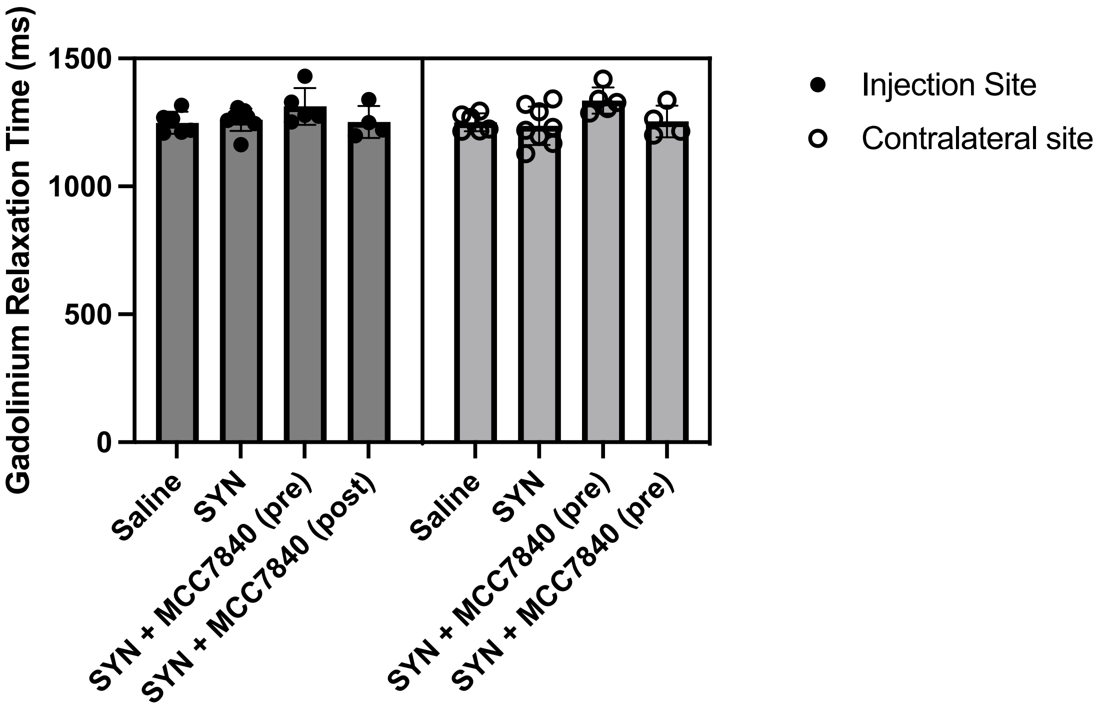

Supplementary Figure 5: Blood brain barrier integrity in the PFF-Syn Parkinson's model. MRI blood-brain-barrier leakage measurement of gadolinium T1 relaxation time values in ipsilateral (injection side) and contralateral hemispheres. Data represent means ± SEM.

Supplementary Figure 6

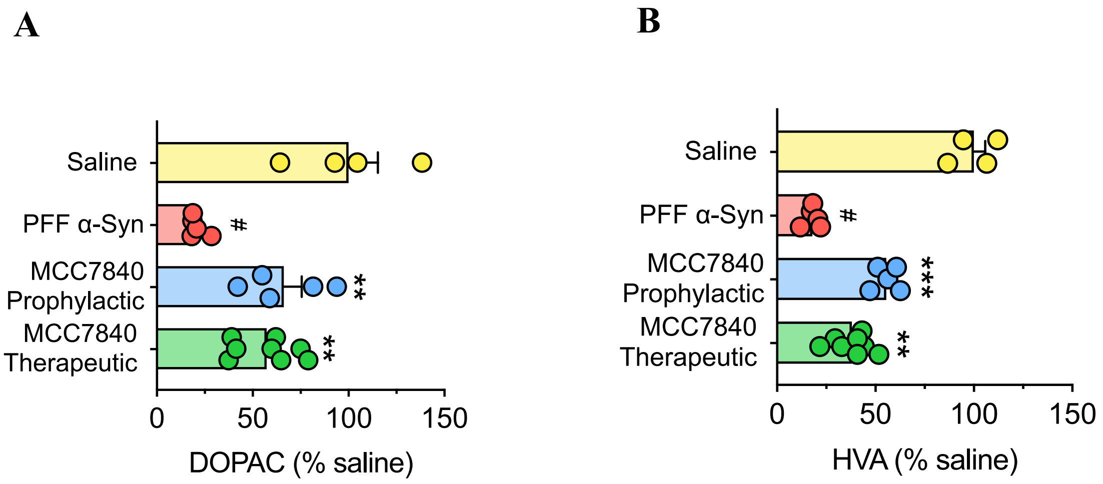

Supplementary Figure 6. Prophylactic or therapeutic oral dosing of MCC7840 ameliorates dopamine degeneration in the PFF-Syn PD mouse model. Mice were injected with PFF-Syn (8 μg) or saline into the striatum and brains were collected after 11 months. Striatal concentrations of [**A**] DOPAC and [**B**] HVA in the groups are shown (n=4-8/group). Data represent means ± SEM, $^{\#}P<0.05$ compared with saline group, $^{**}P<0.01$, $^{***}P<0.001$ compared with PFF-Syn group by one-way ANOVA, and Dunnett's post-test.

Supplementary Table 1

Supplementary Table 1: Drug Molecular Properties of MCC950 and MCC7840

	Drug	MW	tPSA	cLogP	ALogD	HBD	N+O
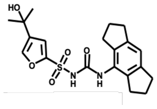	MCC950	404	117	3.4	3.5	3	6
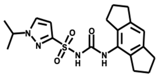	MCC7840	388	101	3.8	4.2	2	7

Supplementary Table 2

Supplementary Table 2: Drug Metabolic Characteristics of MCC950 and MCC7840

Drug	CYP1A2 (μM)	CYP2C9 (μM)	CYP2D6 (μM)	CYP3A4 (μM)	Human LM t½	Mouse LM t½	Human % PPB	Mouse % PPB	Human % PS	Mouse % PS	hERG % inhib
**MCC950**	20	43	>50	>50	>145	>145	99.5	99.4	96	99	21 ± 4.3
**MCC7840**	19	46	>50	>50	>145	>145	99.7	99.5	100	89	4.5 ± 3.1

LM – Liver microsome (half-life in minutes)
PPB – Plasma Protein Binding
PS – Plasma Stability (% remaining at 2 hours)
hERG - human ether-a-go-go K+ ion channel QPatch assay (% inhibition at 30μM)

Supplementary Table 3

Supplementary Table 3: Pharmacokinetic parameters of MCC950 and MCC7840 in C57BL/6J mice

PK Parameters	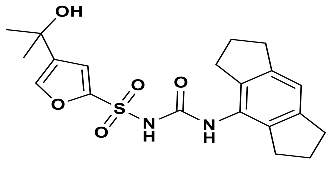	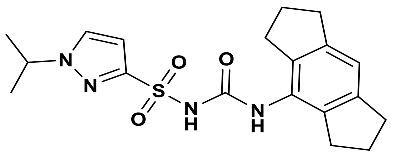
**iv 4 mg/Kg**	**MCC950**	**MCC7840**
C_0 (ng/mL)	**21974**	**52245**
$T_{1/2}$ (h)	**2.00**	**3.39**
Vd_{ss} (L/kg)	**0.306**	**0.160**
Cl (mL/min/kg)	**1.92**	**0.621**
T_{last} (h)	**24.0**	**24.0**
$AUC_{0\text{-}last}$ (ng.h/mL)	**35373**	**107097**
$AUC_{0\text{-}inf}$ (ng.h/mL)	**35381**	**107753**
$MRT_{0\text{-}last}$ (h)	**2.67**	**4.13**
$MRT_{0\text{-}inf}$ (h)	**2.67**	**4.29**
AUC_{Extra} (%)	**0.0216**	**0.614**
$AUMC_{Extra}$ (%)	**0.219**	**4.15**
**po 20 mg/Kg**		
C_{max} (ng/mL)	**34967**	**60467**
T_{max} (h)	**0.333**	**0.250**
$T_{1/2}$ (h)	**2.39**	**5.02**
T_{last} (h)	**24.0**	**24.0**
$AUC_{0\text{-}last}$ (ng·h/mL)	**125422**	**349814**
$AUC_{0\text{-}inf}$ (ng.h/mL)	**125563**	**362035**
$MRT_{0\text{-}last}$ (h)	**3.60**	**6.43**
$MRT_{0\text{-}inf}$ (h)	**3.63**	**7.52**
AUC_{Extra} (%)	**0.117**	**4.21**
$AUMC_{Extra}$ (%)	**0.835**	**15.1**
Bioavailability (%)	**71.0**	**67.2**

Supplementary Table 4

Supplementary Table 4: Pharmacokinetic parameters of MCC7840 following oral administration in multiple species.

Parameter	Mouse	Rat				Dog	Mini-pig	Monkey	
Study number	401321-2015060801-MPK	S-SYG-097	S-SYG-038	S-SYG-057	S-SYG-044	XGS-18	XGS-25	410734-2019091201-CPK	
N= /group	3	3	3	3	3	2	2	3	3
Dose (mg/kg)	20	1	3	10	30	3	3	3	10
T_{max} (hr)[#]	0.25 (0.25)	2.0 (1.0-4.0)	4.0 (2.0-4.0)	4.0 (4.0-8.0)	2.0 (0.08-4.0)	0.75 (0.75)	5.0 (4.0-6.0)	0.5 (0.5-1.0)	0.5 (0.25-0.5)
C_{max} (ng/mL)	60467	2436	6290	31931	89500	21609	6191	17067	86333
C_{max} /dose (ng/mL)/(mg/kg)	3023	2436	2096	3193	2983	7203	2063		
$AUC_{0\text{-}t}$ (ng.hr/mL)	349814	25089	54900	239000	691000	151866	80600	38339	167806
$AUC_{0\text{-}\infty}$ (ng.hr/mL)	362035	27702	56800	244000	696000	161487	109352	38376	167905
$AUC_{0\text{-}t}$ /dose (ng.hr/mL)/(mg/kg)	17491	27702	18933	24400	23200	53829	36450		
$T_{1/2}$ (hr)	5.0	6.5	4.97	4.5	3.2	6.4	11	2.78	2.6
F (%)	67	≥100	≥100	≥100	≥100	83	>62*	≥100	≥100

[#] median (range)

*The extrapolation to determine $AUC_{0\text{-}\infty}$ was >25%, PO AUC_{all} has been used rather than PO AUC_{∞} to determine the bioavailability. The true bioavailability will therefore be greater than this

Abbreviations: N, total Number; T_{max}, time to maximum plasma concentration; C_{max}, maximum plasma concentration; $AUC_{0\text{-}t}$, area under the concentration curve from time zero to last time point; $AUC_{0\text{-}\infty}$, AUC from time zero to infinity; $T_{1/2}$, elimination half-life; F, oral bioavailability; PO, per oral..

Supplementary Table 5

Supplementary Table 5: Extended statistical parameters for all analyses

FIGURE	Test	Degrees of freedom	R squared	P value	F(DFn,DFd)	t,df
Fig1B	log(inhibitor) vs. normalized response	17	0.9667			
Fig1C	log(inhibitor) vs. normalized response	17	0.977			
Fig1D	log(inhibitor) vs. normalized response	35	0.9395			
Fig1E	log(inhibitor) vs. normalized response	35	0.9821			
Fig1F	log(inhibitor) vs. normalized response	11	0.9129			
Fig1G	log(inhibitor) vs. normalized response	11	0.933			
Fig2C	Unpaired t test (two tailed)			0.0325	12.73 ,3, 3)	t=2.768, df=6
Fig3B	Curve comparison Log-rank (Mantel-Cox)	7		<0.0001		
Fig3C	Interpolation	3	0.9958			
Fig3D	Curve comparison Log-rank (Mantel-Cox)	4		<0.0001		
Fig4B	Ordinary one-way ANOVA (Dunnet's multiple comparison)	64	0.631	<0.0001	F (4, 60) = 25.65	
Fig4C	Ordinary one-way ANOVA (Dunnet's multiple comparison)	65	0.6095	<0.0001	F (4, 61) = 23.80	
Fig4D	Ordinary one-way ANOVA (Dunnet's multiple comparison)	65	0.6071	<0.0001	F (4, 61) = 23.57	
Fig4E	Ordinary one-way ANOVA (Dunnet's multiple comparison)	46	0.6707	<0.0001	F (3, 43) = 29.19	
Fig5C	Ordinary one-way ANOVA (Tukey's multiple comparison	29	0.7498	<0.0001	F (2, 27) = 40.45	
Fig5H	Ordinary one-way ANOVA (Dunnet's multiple comparison)	30	0.7549	<0.0001	F (2, 28) = 43.13	
Fig5J	Ordinary one-way ANOVA (Dunnet's multiple comparison)	28	0.8441	<0.0001	F (2, 26) = 70.39	
Fig5L	Ordinary one-way ANOVA (Dunnet's multiple comparison)	28	0.7959	<0.0002	F (2, 26) = 50.68	
Fig5M	Ordinary one-way ANOVA (Dunnet's multiple comparison)	28	0.201	0.0541	F (2, 26) = 3.270	
Fig5N	Ordinary one-way ANOVA (Dunnet's multiple comparison)	28	0.006416	0.9197	F (2, 26) = 0.08395	
Fig50	Ordinary one-way ANOVA (Dunnet's multiple comparison)	28	0.2203	0.0393	F (2, 26) = 3.674	
Fig5P	Ordinary one-way ANOVA (Dunnet's multiple comparison)	29	0.7162	<0.0001	F (2, 27) = 34.07	
Fig6B	Ordinary one-way ANOVA (Tukey's multiple comparison	36	0.5635	<0.0001	F (3, 33) = 14.20	
Fig6C	Ordinary one-way ANOVA (Tukey's multiple comparison	36	0.5595	<0.0001	F (3, 33) = 13.97	
Fig6D	Ordinary one-way ANOVA (Tukey's multiple comparison	35	0.5886	<0.0001	F (3, 32) = 15.26	
Fig6E	Ordinary one-way ANOVA (Tukey's multiple comparison	35	0.5383	<0.0001	F (3, 32) = 12.44	
Fig6F	Ordinary one-way ANOVA (Tukey's multiple comparison	36	0.4508	0.0002	F (3, 33) = 9.029	
Fig6G	Ordinary one-way ANOVA (Tukey's multiple comparison	36	0.6299	<0.0001	F (3, 33) = 18.72	
Fig6H	Ordinary one-way ANOVA (Tukey's multiple comparison	36	0.5661	<0.0001	F (3, 33) = 14.35	
Fig6I	Ordinary one-way ANOVA (Tukey's multiple comparison	36	0.5767	<0.0001	F (3, 33) = 14.98	
Fig6L	Ordinary one-way ANOVA (Tukey's multiple comparison	23	0.748	<0.0001	F (3, 20) = 19.79	
Fig6N	Ordinary one-way ANOVA (Tukey's multiple comparison	39	0.9158	<0.0001	F (3, 36) = 130.5	
Fig6P	Ordinary one-way ANOVA (Dunnet's multiple comparison)	21	0.7426	<0.0001	F (3, 18) = 17.31	
Fig6Q	Ordinary one-way ANOVA (Dunnet's multiple comparison)	18	0.8118	<0.0001	F (3, 15) = 21.57	

Supplementary Material and Methods

Primary mouse microglia cultures

Briefly, postnatal day C57BL/6J mouse pups from day P0 to P1 were collected and washed in Dulbecco's modified Eagle's medium/F-12 nutrient mixture (DMEM-F12, GIBCO Catalogue number-11320) supplemented with 10% heat-inactivated fetal bovine serum (GIBCO), 50 U/mL penicillin, 50 mg/mL streptomycin, 2 mM l-glutamine, 100 mM nonessential amino acids, and 2 mM sodium pyruvate (Invitrogen). Trypsin (Sigma 0.25%) were added to the brains incubated at 37 ^{o}C for 30 min and neutralization of trypsin was done after 30 min by adding an equal amount of media. Mouse brains were triturated and passed through a 70 mm nylon mesh cell strainer. Cells were then incubated in the CO2 incubator at 37 ^{o}C for 14-16 days. The separation of microglia was performed by using a column-free magnetic separation system as previously described [32].

Generation of human monocyte-derived microglia (MDMi)

Briefly, donor buffy coat was diluted 1:1 with phosphate-buffered saline (PBS) and transferred into sterile SepMate 50 (STEMCELL Technologies, BC, Canada) as per manufacturer's instructions. Peripheral blood mononuclear cells (PBMCs) were then collected. Monocytes were positively selected from whole PBMCs using anti CD14+ microbeads (Miltenyi Biotec) and plated at the following densities per well: 1×10^5 cells (96- well plate) and 3×10^5 cells (24-well plate). HMDMs were generated as previously described[34]. To induce the differentiation of MDMi, monocytes were incubated under serum-free conditions using RPMI-1640 Glutamax (Life Technologies) with 1% penicillin/ streptomycin (Lonza) and Fungizone (2.5 μg/ml; Life Technologies) and a mixture of the following human recombinant cytokines: M-CSF (10 ng/ml;Preprotech, 300-25), GM-CSF (10 ng/ml; Preprotech,300-03), NGF-β (10 ng/ml; Preprotech, 450-01), MCP-1(CCL2) (100 ng/ml; Preprotech, 300-04), and IL-34 (100 ng/ml; Preprotech, 200-34-250) under standard humidified culture conditions (37 °C, 5% CO2) for up to 14 days.

Pharmacokinetic analysis

Plasma and brain concentrations of MCC7840 and MCC950 after intravenous or oral administration to fasted 8–10-week-old C57BL/6J male mice was performed using an adapted method as previously described (30). At the relevant time point following dosing, blood samples were collected for plasma and the brain perfused with PBS for 5 min; CSF was also collected from separate groups of mice. Brain homogenates were prepared from dissected brains by homogenizing total brain (0.5 g) with four volumes (2 mL) of deionized water. Samples were analyzed on an AB Sciex 4000QTrap mass spectrometer with 2 Shimadzu Nexera LC-30AD solvent delivery units, Shimadzu Nexera SIL30AC auto-sampler, Shimadzu Prominence DGU-20A5 Degasser, Shimadzu Prominence CBM-20A system controller and Shimadzu Prominence CTO-20A column oven.

Surgical procedure

C57BL/6J mice were anaesthetized using ketamine hydrochloride (100 mg/kg) and xylazine (10 mg/kg), and were placed into a stereotactic frame with nose and ear bars specially adapted for mice. A digital stereotaxic instrument was used with a 5 μL Hamilton syringe to deliver either a sterile saline solution (0.9% sodium chloride) containing 0.2% ascorbic acid, 6-hydroxydopamine (6-OHDA; 12 μg) dissolved in saline containing 0.2% ascorbic acid, α-synuclein pre-formed fibrils (PFF-Syn; 8 μg in PBS), or PBS alone, into the right dorsal striatum of mice at the following stereotaxic coordinates relative to bregma (mm): -2.0 medial-lateral, + 0.5 anterior-posterior, -3.0 mm dorsal-ventral below the pial surface. After drilling a 1 mm burr-hole in the skull, a 2 μl volume of solution was infused at the target site at the rate of 0.5 μl per minute. The needle was held in place for at least 5 minutes after injection to minimize retrograde flow along the needle tract. Mice were given a subcutaneous injection of sterile Ringer's solution to help facilitate recovery and carprofen (5 mg/kg) for post-operative pain relief and were placed on a heat-pad until complete recovery from anaesthesia.

MCC7840 treatment in the 6-OHDA model

8-10 week-old C57BL/6J male mice were housed under a 12-hr light cycle in a SPF climate-controlled facility with food and water provided ad libitum for two weeks prior to study initiation. For MCC7840 treatment in the 6-OHDA model, mice were dosed via oral gavage (1, 3 or 10

mg/kg), or by drinking water (0.3mg/ml), with studies performed at UQ or by CROs. Mice were dosed starting the day before (24 hr prior) stereotaxic surgery, and then daily throughout until sacrifice.

MCC7840 administration as prophylactic and therapeutic in the PFF-Synuclein mouse model

8-10 week-old C57BL/6J male mice were housed under a 12-hr light cycle in a SPF climate-controlled facility with food and water provided ad libitum for two weeks prior to study initiation. MCC7840 (or water alone for control animals) was administrated to mice in drinking water ad libitum at 0.3mg/ml. For prophylactic MCC7840 dosing, treatment commenced one day before PFF-Syn injection. For therapeutic dosing, treatment commenced 4 months after disease induction with PFF-Syn.

Blood collection and IL-1β plasma determination

Blood samples were taken, and plasma prepared using Vacuette Minicollect 0.5 ml EDTA tubes (Greiner, catalogue #450530) according to the manufacturer's instructions. Plasma was aliquoted immediately and stored in aliquots at -80 °C until further analysis. IL-1β concentrations in diluted mouse plasma samples was measured using the mouse IL-1β kit (R&D Systems, Catalogue # DY008).

Behavioural test

All behavioural tests were performed during the light phase of the light/dark cycle. Prior to each test, the mice were moved to the testing room for an acclimatization period of at least 30 min. Instruments and tools used for the behavioural tests were cleaned thoroughly with 70% ethanol and rinsed with sterile water between trials to minimize odours.

Amphetamine induced rotations

Amphetamine-induced ipsilateral rotations were performed in the 6-OHDA model. Mice were injected with 2 mg/kg of D-amphetamine and placed in circular glass bowls. After an acclimatization period of 5 min, the net ipsilateral rotations over 10 min were recorded and counted. Quantitation was performed from recorded videos by an investigator blinded to the treatment groups.

Balance beam test

Mice were tested on a 0.5 cm wide, 1 m long balance beam apparatus. The balance beam consisted of a transparent Plexiglas structure that was 50 cm high with a dark resting box at the end of the runway. Mice were trained on the beam for three times in the morning, allowing for a resting inter-trial period of at least 15 min. Mice were left in the dark resting box for at least 10 s before being placed back in their home cage. Mice were then re-tested in the afternoon, at least 2 hr after the training session. During test sessions, mice performance was recorded. The test consisted of three trials with a resting inter-trial period of at least 10 min. The latency to cross the beam was recorded for the last of the three tests. Mice were tested, at 4, 6, 8 and 10 months after PFF-Syn or vehicle injection.

Rotarod test

The accelerated rotarod test was performed over 3 consecutive days allowing for 2 days of training and acclimatization [18]. Three trials per day were performed using a Rotarod (Ugo Basile) apparatus with an accelerated speed of 5–40 RMP in 5 min. A resting time of at least 30 min was given between trials. Latency to fall was recorded at each time. Every mouse able to stay on the rotating rod for more than 5 min was removed and its latency recorded as 300 s. The average of the 3 trials performed is presented. Mice were tested, at 4, 6, 8 and 10 months after PFF-Syn or vehicle injection

Radiotracer synthesis

The tosylate precursor for [^{18}F]DPA-714 and the reference standard were made according to literature procedures [39]. The mesylate precursor (MBCTT) and the reference standard used for the synthesis of [^{18}F]FBCTT were obtained from ABX. The reagents, precursor and reference standards used for the synthesis of [^{18}F]FDOPA were supplied by ABX. [^{18}F]DPA-714 was synthesised by a modified procedure previously described by James *et al.*[37]. [^{18}F]FBCTT (also known as [^{18}F]PR04.MZ) was synthesised using a procedure previously described by Riss *et al.*[38]. [^{18}F]FDOPA was synthesised by using an adapted protocol to that discussed directly with the supplier (ABX).

Radiosynthesis of [^{18}F]DPA-714

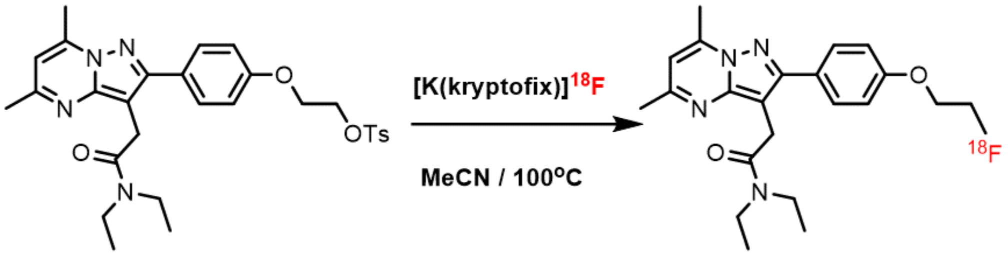

Scheme 1: Radiosynthesis of [^{18}F]DPA-714**.**

[^{18}F]DPA-714 was synthesized by nucleophilic fluorination of the tosylate precursor with [^{18}F]fluoride (17 to 20 GBq) on a Synthra RN-Plus (Scheme 1).

[^{18}F]Fluoride in oxygen-18 enriched water (98%) was transferred using helium pressure to the hot-cell where it was measured in a dose calibrator and then transferred into the "V-vial" of the Synthra RN_{Plus} synthesis module. The aqueous [^{18}F]F^- was transferred under vacuum to the QMA cartridge, with the enriched water directed to a $H_2{}^{18}O$ recovery vial. The [^{18}F]F^- was eluted from the cartridge and collected in the glassy carbon reaction vessel using an eluent solution of Kryptofix® and K_2CO_3. Drying of the fluoride was performed under helium flow with vacuum applied at a temperature of 68°C for 5 minutes, then at 95°C for a further 10 minutes. Once the helium flow had ceased and the reaction vessel had cooled to 30°C then the vacuum was released. A temperature of 30°C was employed to ensure that there was no loss of the solvent added in the subsequent step. The tosylate precursor in MeCN was added into the reaction vessel and radiolabelling was conducted at 100°C. The reaction vessel was then cooled, and water added. The diluted crude reaction mixture was purified by HPLC. Semi-prep HPLC purification was achieved under isocratic conditions using an Eclipse XDB-C18 Semi-Prep 5µm (9.4 mm × 250 mm; p/n 990967-202). Mobile phase: 45% MeCN / 55% 0.1 M NH_4OAc with a flow rate of 3 mL/min (the desired product eluted with a retention time of ca. 21 minutes).

The purified product was cut to a collection vial containing 60 mL of water and stirred. Helium pressure was applied to the vial to transfer the contents through a manually conditioned (5 mL EtOH, 5 mL water then 10 mL air) C18 cartridge (Waters Sep-Pak® C18 Plus). The loaded cartridge was then washed with 10 mL, and then dried using helium flow. [^{18}F]DPA-714 was eluted from the C18 cartridge with 0.5 mL EtOH to the vented product collection vial containing

4.5 mL 0.9% saline with a non-corrected radiochemical yield of 17 ± 4 % (n=10) with a molar activity of 200 ± 46 GBq / µmol. '

Quality control of [^{18}F]DPA-714

The radiochemical purity of the product was determined by HPLC and was >99% for at least 4 hours. [^{18}F]DPA-714 has a retention time of 7.9 minutes and co-elutes with the reference standard DPA-714.

For all productions, the analytical column was measured in a dose calibrator immediately after the analysis, which revealed no residual activity was left on the analytical column.

Analytical HPLC for [^{18}F]DPA-714 used an Eclipse Plus C18 column, 5 µm (4.6 × 150 mm, Agilent, Part NO. 959993-902) using 0.1M ammonium acetate as solvent A and MeCN as solvent B at a flow rate of 1 mL/ min. The method for the analysis was 0 to 20 min (45% B), 20 to 21 min (45 to 95% B), 21 to 26 min (95% B), 26 to 27 min (95 to 45% B), 27 to 30 min (45% B).

Radiosynthesis of [^{18}F]FBCTT

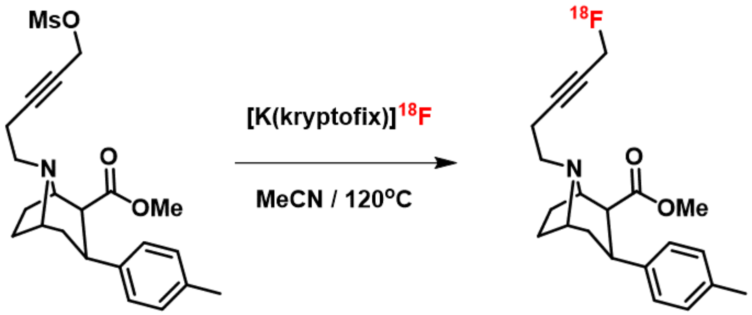

Scheme 2: Radiosynthesis of [^{18}F]FBCTT**.**

[^{18}F]FBCTT was synthesized by nucleophilic fluorination of the mesylate precursor with [^{18}F]fluoride (17 to 21 GBq) on a Synthra RN-Plus (Scheme 2).

[^{18}F]Fluoride in oxygen-18 enriched water (98%) was transferred using helium pressure to the hot-cell where it was measured in a dose calibrator and then transferred into the "V-vial" of the Synthra RN_{Plus} synthesis module. The aqueous [^{18}F]F^- was transferred under vacuum to the QMA cartridge, with the enriched water directed to a $H_2{}^{18}O$ recovery vial. The [^{18}F]F^- was eluted from the cartridge

and collected in the glassy carbon reaction vessel using an eluent solution of Kryptofix® in 0.5 mL MeCN and K_2CO_3 in 0.5 mL water. Drying of the fluoride was performed under helium flow with vacuum applied at a temperature of 68°C for 5 minutes, then at 95°C for a further 10 minutes. Helium flow ceases and the reaction vessel was cooled to 30°C before the vacuum was stopped. A temperature of 30°C was employed to ensure that there was no loss of the solvent added in the subsequent step. The mesylate precursor (0.5 to 0.6 mg, 1.2 to 1.4 µmol) dissolved in 1 mL MeCN was added into the reaction vessel and radiolabelling was conducted at 120°C for 10 minutes whilst stirring. The reaction vessel was then cooled and 1 mL water was added. The diluted crude reaction mixture was purified by HPLC. Semi-prep HPLC purification was achieved under isocratic conditions using an Eclipse XDB-C18 Semi-Prep 5µm (9.4 mm × 250 mm; p/n 990967-202). Mobile phase: 60% MeCN / 40% 0.1 M NH_4OAc with a flow rate of 4 mL/min (the desired product eluted with a retention time of approx 19 minutes). The purified product was cut to a collection vial 80 mL of water and stirred. Helium pressure was applied to the vial to transfer the contents through a manually conditioned (5 mL EtOH, 5 mL water then 10 mL air) C18 cartridge (Waters Sep-Pak® C18 Plus 46 mg). [^{18}F]FBCTT was eluted from the C18 cartridge with 1 mL EtOH to the vented product collection vial containing 9 mL 0.9% saline (with 100 mg of L-ascorbic acid) with a non-corrected radiochemical yield of 17 ± 4 % (n=10) with a molar activity of 205 ± 48 GBq / µmol.

Quality control of [^{18}F]FBCTT

The radiochemical purity of the product was determined by HPLC and was >99% for at least 4 hours. [^{18}F]FBCTT has a retention time of 10.8 minutes and co-elutes with the reference standard FBCTT.

For all productions, the analytical column was measured in a dose calibrator immediately after the analysis, which revealed no residual activity was left on the analytical column.

Analytical HPLC for [^{18}F]FBCTT used an Eclipse Plus C18 column, 5 µm (4.6 × 150 mm, Agilent, Part NO. 959993-902) using 0.1M ammonium acetate as solvent A and MeCN as solvent B at a flow rate of 1 mL/ min. The method for the analysis was 0 to 20 min (60% B), 20 to 21 min (60 to 95% B), 21 to 26 min (95% B), 26 to 27 min (95 to 60% B), 27 to 30 min (60% B).

Radiosynthesis of [^{18}F]FDOPA

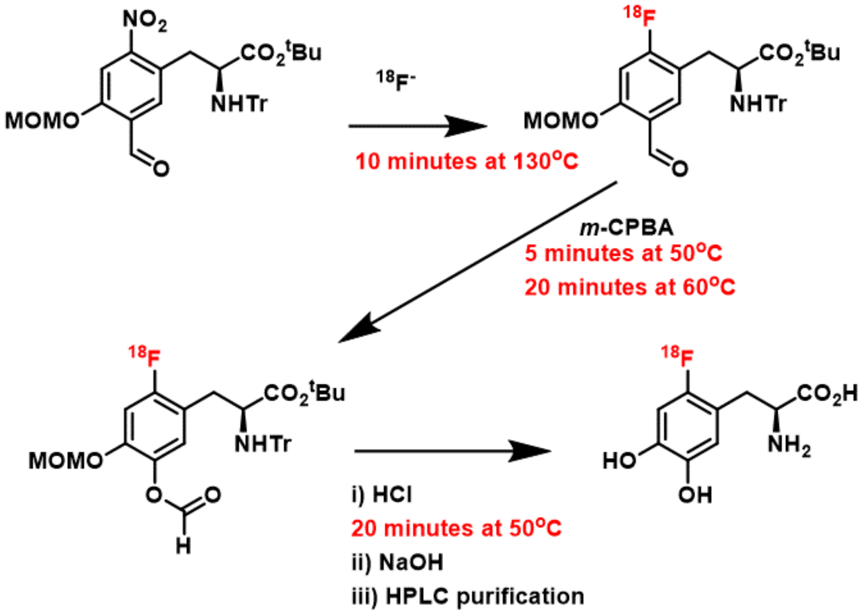

Scheme 3: Radiosynthesis of [^{18}F]FDOPA**.**

[^{18}F]F-DOPA was synthesized by in three steps from the aldehyde precursor in Scheme 3 from [^{18}F]fluoride (38 to 41 GBq) on a Synthra RN-Plus.

[^{18}F]Fluoride in oxygen-18 enriched water (98%) was transferred using helium pressure to the hot-cell where it was measured in a dose calibrator and then transferred into the "V-vial" of the Synthra RN_{Plus} synthesis module. The aqueous [^{18}F]F^- was transferred under vacuum to the QMA cartridge, with the enriched water directed to a $H_2{}^{18}O$ recovery vial. The [^{18}F]F^- was eluted from the cartridge and collected in the glassy carbon reaction vessel using an eluent solution of Kryptofix® in 0.5 mL MeCN and K_2CO_3 in 0.5 mL water. Drying of the fluoride was performed under helium flow with vacuum applied at a temperature of 68°C for 5 minutes, then at 95°C for a further 10 minutes. Helium flow ceases and the reaction vessel was cooled to 30°C before the vacuum was stopped. A temperature of 30°C was employed to ensure that there was no loss of the solvent added in the subsequent step. The aldehyde precursor (10 mg, 29.5 µmol) dissolved in 1 mL DMSO was added into the reaction vessel and radiolabelling was conducted at 130°C for 10 minutes whilst stirring. The reaction vessel was then cooled and 3 mL of a 30% MeCN / 70 % water solution was added. The crude F-benzaldeyde derivative reaction mixture was trapped on a C18ec cartridge (previously conditioned with 10 mL MeCN, 10 mL water and 20 mL air). The reaction vessel was washed with

an additional 3 mL of a 30% MeCN / 70 % water solution which was then also passed through the C18ec cartridge. The cartridge was washed with further aliquots of a 30% MeCN / 70% water solution (2 × 12 mL) and then dried for 2 minutes with helium. Multiple washings with 30% MeCN / 70 % water solution were performed to (i) remove unreacted [^{18}F]fluoride, (ii) the potassium kryptofix carbonate complex and (iii) all traces of DMSO. The F-benzaldehyde derivative was eluted into the second reaction vessel using a solution containing *m*-chloroperbenzoic acid (approx. 7 mg, 40 µmol) in 2 mL of MeCN. The acetonitrile was removed under evaporation (and vacuum) initially at 50°C for 5 minutes and then at 60°C for 20 minutes (during which time the product had oxidised). After cooling, deprotection was achieved by addition of 30% HCl (1 mL) and EtOH (0.1 mL) with heating (and stirring) at 50°C for 20 minutes. After cooling, the solution was neutralised by addition of NaOH (4 M, 1.9 mL). The crude solution was passed through an alumina N cartridge (conditioned with 10 mL water and 10 mL air) and loaded onto the HPLC loop. Purification was performed using a phosphate buffer (pH 6.3) containing Na-EDTA (approx. 1.5 mg / mL of buffer) and L-ascorbic acid (10 mg / mL of buffer) using a Waters SunFire C18 OBD column (100 × 250 mm, Waters, Part NO. 186008155). The product elutes with a retention time of approx. 9 minutes.

Quality control of [^{18}F]FDOPA

The radiochemical purity of the product was determined by HPLC using a C18 reverse phase column and was >98% for at least 4 hours. The analytical column was measured in a dose calibrator immediately after the analysis of all productions, which revealed less 1% residual activity was left on the analytical column. [^{18}F]FDOPA elutes with a retention time of 5.7 minutes and co-elutes with F-*L*-DOPA. The observed radiochemical impurity (approx. 1%) was a radiolysis product which had a retention time of 7.8 minutes. Analytical HPLC for [^{18}F]FDOPA using the reverse phase C18 system using an XTERRA RP18 , 5 µm (4.6 × 250 mm, Waters, part no. 186000496) using 0.8% TFA in water as solvent A and MeCN as solvent B at a flow rate of 1 mL/ min. The method for the analysis was 0 to 10 min (10% B), 10 to 15 min (10 to 90% B), 15 to 20 min (90% B), 20 to 22 min (90 to 10% B), 22 to 26 min (10% B) at a UV wavelength = 283 nm. Chirality of the [^{18}F]FDOPA was determined using a chiral column (Crownpak R+, 4 x 150 mm, p/n 27714) and revealed 94% [^{18}F]-*L*-DOPA (retention time of 6.6 minutes), 6% [^{18}F]-*D*-DOPA (retention time of 5.1 minutes) with an enantiomeric excess of 88%. Co-elution was performed using both F-*L*-

DOPA and F-*rac*-DOPA. Chiral HPLC method: Daicel Crownpak CR+ (4.0 × 150mm x 5µm, Part No. 27714), Flow rate = 0.8ml/min with 100% (perchloric acid pH 2.0).

PET/MRI imaging in the 6-OHDA model

Twenty-four days post-surgery, mice from each experimental group were injected with a radiotracer every second day in a group of 3-4 mice per imaging week. Three different radiotracers were used on three different days. [^{18}F]DPA-714 (24th day post-surgery) was used to target the translocator protein, TSPO, overexpressed in activated microglia during neuroinflammation. [^{18}F]FBCTT (26th day post-surgery) was used to estimate the expression of functional dopamine transporter and [^{18}F]FDOPA (28th day post-surgery) to study the presynaptic dopamine function. For the first imaging session, Gadovist, a Gadolinium based MRI contrast agent routinely used in clinical and preclinical imaging, was added to the injection solution to visualize the blood brain barrier integrity.

Anaesthetised mice, with a cannulated tail vein, (0.010”× 0.030”OD Tygon microbore tubing, 55 cm long, and 30 gauge needles) were placed in a combined MRI/PET system, comprising a 300 mm bore 7T ClinScan, running Siemens VB17, and removable PET insert containing 3 rings of 16 detector blocks with 15×5 LSO crystals (1.6 × 1.6 × 10mm) per block, at the center of the magnet bore operating under Siemens Inveon Acquisition Workplace (IAW) (Bruker, Germany). A 23 mm ID mouse head MRI rf coil inside the PET ring was used to acquire mouse head images simultaneously with the PET acquisition. Mice were maintained under 1% to 2% isoflurane in an air-oxygen mixture at a flow rate of 1 L/min for the duration of the imaging session and monitored by a breathing pillow.

[^{18}F]DPA-714 imaging

On Day 24 post-surgery, mice (25.6 ± 1.62 g, n=30) were injected with a mixture of [^{18}F]DPA-714 (9.47 ± 2.02 MBq, n=30) and Gadovist (50µl) in saline/10% ethanol in a total volume of 200µl. Following acquisition of fast localizer images, a 3D VIBE T1 weighted sequence was acquired, and 5 dynamic transverse slices were planned. The PET acquisition (60 min) and dynamic images (13 min) were then started simultaneously. Following 2 min baseline imaging the PET and MR agent mixture was injected. Following the dynamic MR images, T2 weighted anatomical images, 3D VIBE T1 weighted sequence, 3D VIBE T1map, and short repeats of the

dynamic scanning were acquired during the 60 min PET scan. Following scanning, the activity in the cannulation line was measured to enable calculation of the injected dose.

[^{18}F]FBCTT imaging

On Day 26 post-surgery, the same mice were injected with [^{18}F]FBCTT (6.84 ± 1.08 MBq, n=29) in saline/10% ethanol/10mg/mL of L-ascorbic acid in a total volume of 200µl. The cannulated mouse was placed in the MR-PET scanner and positioning checked with fast localiser imaging. A 60 min PET acquisition was started and following 2 min baseline the PET tracer was injected. During the PET acquisition, T2 weighted anatomical images, 3D VIBE T1map and T2 multi-contrast T2 map sequences acquired. Following the scanning the activity in the cannulation line was measured to enable calculation of the injected dose.

[^{18}F]FDOPA imaging

On Day 28 post-surgery, a COMT inhibitor Entacapone (25mg/kg, Sigma) and a AADC inhibitor (Benserazide, 10mg/kg, Sigma) were given by intraperitoneal injection 45 min and 30 min, respectively, before the administration of [^{18}F]FDOPA to improve the availability of FDOPA in mouse brain. The mice were injected with [^{18}F]FDOPA (8.58 ± 2.42 MBq, n=29) in Phosphate buffer pH=6.3 /10mg/mL of L-ascorbic acid in a total volume of 200 µL. The cannulated mouse was placed in the MR-PET scanner and positioning checked with fast localiser imaging. A 90 min PET acquisition was started and following 2 min baseline the PET tracer was injected. During the PET acquisition, T2 weighted anatomical images, 3D VIBE T1map, 3D susceptibility images and T2 weighted images with and without magnetisation transfer contrast were acquired. Following the scanning the activity in the cannulation line was measured to enable calculation of the injected dose

PET image reconstruction and analysis

The list-mode data were sorted into 20 dynamic frames (10 x 60 sec, 10 x 300 sec time frames) for [^{18}F] DPA-174 and [^{18}F]FBCTT and into 26 dynamic frames (10 × 60 sec, 16 × 300 sec time frames) for [^{18}F]FDOPA. The resulting sinograms were reconstructed with a two dimensional ordered-subset expectation maximization (OSEM2D) algorithm. The dynamic PET images were registered and displayed with the T2-weighted MRI images using Siemens Inveon Research Workplace (IRW version 4.1, Siemens). The T2-weighted MRI images, display excellent

anatomical resolution and contrast, were used to delineate region of interests (ROIs) throughout the brain.

The striatum was the ROI analysed on the lesioned and non-lesioned sides of the brain. For each PET image, three-dimensional ellipsoidal regions of interest (ROIs) were manually defined to the saline or 6-OHDA lesioned site in the right striatum region (Ipsilateral Striatum) under the guidance of T2-weighted MR images using the IRW 4.1 software. Another ROI with identical shape was drawn in the left (contralateral) striatum. Activity per voxel was converted to Bq/mL using a conversion factor obtained by scanning a cylindrical phantom filled with a known activity of 18F to account for PET scanner efficiency. The mean value in each ROI was used to generate regional time activity curves (TACs). Individual TACs were normalized by the injected dose and the animal weight, and then expressed as standardized uptake value (SUVs). The standardised uptake value ratio (SUVR) was calculated by dividing the SUV of the ipsilateral striatum by that of the contralateral striatum.

MRI data analysis

MRI images were analysed using HOROS DICOM viewer. The T2 weighted MR images used to define PET ROI were also used to manually define the striatum for MRI analysis. The ROI of the striatum was saved and then applied to the T1 maps acquired after the Gadovist injection on day 1. The mean T1 time (msec) was reported for the ipsilateral and contralateral striatum for each mouse.

PET/MRI imaging and Quantification in the PFF-Syn model

Anaesthetised mice, with a cannulated tail vein, (0.010”× 0.030”OD Tygon microbore tubing, 55 cm length, 30-gauge needle), were placed in a combined MRI/PET system comprising a 300 mm bore 7T ClinScan, (Siemens VB17) and a removable PET insert containing 3 rings of 16 detector blocks, each containing 15 x 15 LSO crystals (1.6 x 1.6 x 10mm) located at the magnet isocentre. Data were acquired using Siemens Inveon Acquisition Workplace (IAW, Bruker, Germany). A 23 mm ID mouse head MRI RF coil, positioned inside the PET ring, enabled simultaneous PET and MRI acquisition.

Mice were maintained under 1-2% isoflurane in an air-oxygen mixture (1L/min) for the duration of the imaging session and monitored by a breathing pillow. Following acquisition of localiser

images, dynamic gradient echo imaging and the PET scanning were initiated simultaneously for a total duration of 60 min. After 2 min of baseline acquisition, mice were injected via the tail vein with a mixture of 18F-DPA-714 (13.32 ± 2.44 MBq, mean ± SD) and Gadovist (50µl) in saline/10% ethanol in a total volume of 200µl. Gadovist, a gadolinium-based MRI contrast agent, routinely used in clinical and preclinical imaging, allowing to visualise the blood brain barrier integrity.

Thirteen minutes of dynamic PET/MRI data were acquired before anatomical MRI sequences, including T2-weighted images, 3D VIBE T1-weighted images, and T1 maps. Short repeats of dynamic PET acquisition were interleaved with MRI sequences over the remainder of the 60 min session.

List-mode PET data were reconstructed as above and co-registered to each animal's T2-weighted MRI. The striatum was selected as the primary target region for quantification. For each animal, a three-dimensional ellipsoidal ROI of fixed voxel dimensions and orientation was manually placed on the ipsilateral striatum using MRI guidance; the contralateral ROI was mirrored to the exact coordinates in the opposite hemisphere to ensure identical size, shape, and placement. ROI placement was based on MRI landmarks (distance from bregma, ventricle boundaries, cortical surface) to standardise anatomical correspondence across animals. All ROIs were manually drawn by a single trained operator blinded to experimental group, with independent verification by a second operator. No intensity-based thresholding was applied; all ROIs were anatomically defined.

Mean activity concentration (Bq/mL) in each ROI was converted to SUV by normalising to injected dose and body weight:

$$SUV = \frac{\text{ROI activity concentration (Bq/mL)}}{\text{Injected activity (Bq) / body weight (g)}}$$

The SUVR was calculated as:

$$SUVR = \frac{\text{SUV}_{\text{ipsilateral striatum}}}{}$$

$$\overline{SUV_{contralateral\ striatum}}$$

TACs were generated from SUV data for each region. For MRI analysis, the same anatomically defined ROIs used for PET quantification were applied to T1 maps, and mean T1 relaxation times (ms) were extracted for each hemisphere.

Ex vivo Autoradiography

Mice were injected with [^{18}F]DPA-714 (25.4 ± 6.96 MBq, mean ± SD) via a tail vein catheter under anaesthesia (2.5 % isoflurane in O2 at 1 l/min flow rate) and were kept awake in individual cages until they were sacrificed by decapitation at 45 min (n = 2-3/group) post-tracer injection. Brain were removed and rapidly frozen in methylbutane (Fisher Scientific, UK) on dry ice (between −42 and −44 °C). Coronal sections (20 µm) mounted on adhesive microscope slides (Superfrost Plus, Thermo Scientific, Germany) were obtained using a cryotome (CM3050S, Leica, Germany), dried and exposed to a phosphor storage screen film (BAS-IP MS 2040, FujiFilm, Japan) overnight. The screens were read using an Amersham Typhoon Biomolecular Imager (GE Healthcare, USA) and analyzed using ImageQuant TL image analysis software from GE Healthcare. To further demonstrate the binding specificity of [^{18}F]DPA-714, we performed *ex vivo* blocking study. Mice were first injected i.v. with unlabelled PK11195 (1 mg/kg), a specific high-affinity ligand to the mitochondrial TSPO. Five minutes later, [^{18}F]DPA-714 was administered also through tail vein injection. After 45 min, the mice were sacrificed, and the brain were removed and sectioned as described in the section above.

For the quantification, regions-of-interest (ROIs) were drawn on the brain images from each mouse (n=8-10 brain sections were used to draw ROIs from each of the injection site and the contralateral site) using an irregular shaped drawing tool, to measure the amount of radioactivity in each of the brain sections. Similar ROIs were drawn in a background region (n=10 regions) to enable background subtraction. The results were expressed as digital light units per square millimetre normalized for body weight of the animal and injected dose [(DLU/mm2) × (body weight/injected dose)].

LC-MS/MS quantification of striatal dopamine and metabolites

Mice were sacrificed and striatal tissue was micro-dissected, weighed and snap-frozen at -80 °C. Neurotransmitters were extracted and derivatized using ethyl chloroformate. Striatal dopamine (DA) and its metabolites (DOPAC and HVA) were quantified in their stable derivative form in the presence of internal standard 3, 4-dihydroxybenzylamine (DHBA) using highly sensitive liquid chromatography-tandem mass spectrometry (LC-MS/MS) as described previously [39]. An API 3200 (AB SCIEX) triple quadrupole Q TRAP LC/MS/MS system was used with Turbo V ion source coupled with Agilent series HPLC system under positive (+1) ionization in multi-reaction mode. Samples were chromatographed on a Phenomenex synergi fusion – RP 80 Å analytical column (150 × 4.6 mm; 4 µm) under a binary gradient condition at 500 µl flow rate using mobile phase A (0.1% formic acid in milliQ water) and mobile phase B (0.1% formic acid in acetonitrile). For quantitation, one transition per analyte was monitored and two transitions per analyte were monitored for qualitative purposes.

Immunofluorescence studies

Coronal sections (20 µm), previously obtained for autoradiography experiments, were post-fixed in 4% paraformaldehyde (PFA) for 30 minutes at room temperature (RT), and processed for immunofluorescence as previously described[26]. Sections were blocked with PBS containing 2% bovine serum albumin (BSA), 2% normal donkey serum, 0.1% Triton X-100 and 0.05% Tween-20 for 2 h at room temperature. Sections were incubated overnight at RT with the following primary antibodies diluted in PBS containing 2% BSA and 2% donkey serum: Iba1 (Cat# 019-19741, Wako); or GFAP (Cat# 13-0300, Thermo Fisher). After five washes in PBS, sections were incubated with Alexa fluor-conjugated secondary antibodies for 2h at RT. Nuclei were counterstained with DAPI, and slides were mounted using fluorescence mounting medium (Dako) according to manufacturer's instructions. Images were acquired using a Zeiss AxioScan Z1 Fluorescent slide scanner.

Author Contributions

TW and MC designed and led the project. EA helped design and performed most of the experiments. KM, RB, GC, CC, VK, DS, MB, RG, RC, KS, RP, RH, AM, KM, and AR, contributed to acquisition, analysis or interpretation of data. AR contributed to MCC950 and MCC7840 synthesis. EA performed *in vitro* experiments, and *in vivo* MWS and Parkinson's disease models. MB, CC, RP, RG and VK contributed to pharmacokinetic studies. RB and DS contributed to radiotracer synthesis, and EA, KM, and GC to PET/MRI imaging. TW and EA analysed the final data and generated the figures. EA, MC, and TW wrote the manuscript. All authors approved the final version of this article.
